# Rapid functionalization of single-walled carbon nanotubes in polar solvents

**DOI:** 10.1126/sciadv.aeb3240

**Published:** 2026-09-11

**Authors:** Dominik Just, Ryszard Siedlecki, Maciej Krzywiecki, Oussama Er-Riyahi, Yann Pouillon, Javier Junquera, Karolina Z. Milowska, Dawid Janas

**Affiliations:** ^1^ Department of Chemistry, Silesian University of Technology, B. Krzywoustego 4, 44-100 Gliwice, Poland.; ^2^ Institute of Physics—Centre for Science and Education, Silesian University of Technology, Konarskiego 22B, 44-100 Gliwice, Poland.; ^3^ CIC nanoGUNE, Donostia-San Sebastián 20018, Spain.; ^4^ Departamento de Ciencias de la Tierra y Física de la Materia Condensada, Universidad de Cantabria, Avenida de los Castros, s/n, Santander E-39005, Spain.; ^5^ Ikerbasque, Basque Foundation for Science, Bilbao 48013, Spain.

## Abstract

Fluorescent semiconducting single-walled carbon nanotubes (SWCNTs) hold considerable promise for photonics. Furthermore, their optical characteristics can be improved by functionalization, which generates additional spectral features. However, the importance of the solvent in which the SWCNT modification is conducted remains underexplored. In this work, the complex relationships between solvent, dispersant, and SWCNTs were untangled to unravel the underlying phenomena. Through a systematic investigation of SWCNT reactivity in a broad spectrum of solvents, supported by multiscale modeling enabled by our implementation of a hybrid functional within SIESTA, we found that the solvent medium and the dispersant enabling SWCNT solubilization affect not only the kinetics but also the course of the modification of SWCNTs. Polar solvents induced notable structural reorganization of polymers on the SWCNT surface and enhanced charge redistribution at the polymer-SWCNT interface. Consequently, we achieved control over the optical properties of SWCNTs, and the tailored SWCNTs enabled optical detection of cholesterol, a substantial risk factor for cardiovascular diseases.

## INTRODUCTION

Single-walled carbon nanotubes (SWCNTs) have unique properties that captivate scientists, specifically in the context of photonics (*1*–*5*). In particular, the sensitivity of SWCNTs’ optical properties to the surrounding environment makes them promising for sensing a broad spectrum of analytes such as dopamine (*6*–*8*), serotonin (*7*), and cholesterol (*9*), among other applications (*10*–*13*). However, the problem is that the photoluminescence quantum yield (PLQY) of these materials is unsatisfactorily low, hindering their utilization as sensors. Fortunately, light emission resulting from the radiative recombination of excitons (electron-hole pairs) (*3*) can be greatly improved by chemical modification of SWCNTs (*4*, *14*–*18*). Consequently, the mobile excitons may be trapped at the as-generated defect sites, which function as potential energy wells. This approach considerably enhances the photonic characteristics of SWCNTs. First, the PLQYs of SWCNTs grafted with luminescent defects are often higher than those of pristine SWCNTs (*3*, *4*, *17*, *19*). Second, the confinement of excitons creates additional red-shifted PL bands, the emission of which is even better aligned with the transparency window of tissue. Therefore, optimizing the number and type of introduced luminescent defects to the SWCNT surface is essential.

These luminescent defects [also referred to as quantum defects or organic color centers (OCCs)] occur in various forms: (i) oxygen-based functional groups (*9*, *18*, *20*, *21*), (ii) aliphatic substituents (*14*, *16*), and (iii) aromatic moieties (*14*, *16*, *22*–*25*). Several synthetic strategies have been designed to implant them, which give some degree of control over the optical emission characteristics of the covalently modified SWCNTs (*3*, *4*, *15*, *16*). In addition to the aforementioned biomedical applications, which require near-infrared (NIR) emission, the PL at ∼1300 nm would be especially desirable. This way, the modified SWCNTs emitting in this range would also hold considerable value for telecommunication, as the 1300-nm wavelength is among the most efficient bands for optical information technology (*26*). Unfortunately, reaching this goal, especially using the most abundant (6,5) SWCNTs, is challenging. Commonly, the *E*_11_^*-^ (also called *E*_11_^2*^) peak positioned in this range emerges at an overly high SWCNT defect density, resulting in the deterioration of the optical properties of the material, such as PLQY (*3*, *4*, *15*, *16*). Shiraki *et al.* obtained SWCNTs emitting in the vicinity of this range by using divalent bisdiazonium compounds (*27*). This reactant was able to attach to two relatively close locations on the SWCNT, sufficiently disturbing the exciton recombination characteristics to obtain PL emission at ∼1250 nm. A similar concept was explored by Maeda *et al.*, who used 1,4-diiodooctafluorobutane, a compound containing two reactive sites (*28*). Due to two SWCNT attachment points and the presence of strongly electron-withdrawing fluorine substituents, the PL emission was registered at 1320 nm. Furthermore, Settele and co-workers observed that 2-iodoaniline, when used in the dark and attached to SWCNTs, also produced an additional spectral feature at ∼1250 nm (*23*). This result likely originated from the dual attachment of 2-iodoaniline to SWCNTs. The importance of the configuration of the functional group added to the SWCNTs was confirmed by Wang *et al.*, who revealed that not only the character of the aryl substituent, but also the chemical identity of the pairing group matters (*29*). The use of various nucleophilic compounds that produce the pairing group can shift the PL emission band of (6,5) SWCNTs to nearly 1300 nm. Recently, Yu and colleagues (*25*) as well as Maeda and his team (*30*) reported that the steric hindrance introduced by the substituent can also affect the electronic/optical properties of the SWCNTs to a sufficient extent, so that the additional PL peaks generated can be observed in the discussed spectral range. Last but not the least, Espinoza *et al.* recently disclosed that the use of sodium hypochlorite under high pH conditions created a peak at 1230 nm (*31*). Despite the merits of these studies, the current understanding of the mechanism by which luminescent defects are introduced to SWCNT is still incomplete. This knowledge gap is partially caused by the lack of comprehension of how the solvent environment and dispersant molecules affect the nature of SWCNTs as well as their propensity for chemical modification.

The impact of the solvent choice is rarely considered. Most commonly, SWCNTs are functionalized in water, which is an easy-to-handle medium, using reactive oxygen species (*7*, *9*, *20*, *31*, *32*) or diazonium chemistry (*15*, *22*, *24*, *33*). Regarding organic solvents, which are less popular, two approaches dominate the field: either SWCNTs are processed in toluene to implant an aryl group onto the surface (*23*, *24*), or they are subjected to reductive alkylation in anhydrous tetrahydrofuran (THF) (*16*, *30*). Nonetheless, the state of the art lacks a detailed analysis of the impact of the solvent on the course of SWCNT functionalization. In this context, scientists have so far studied the influence of the solvent environment on the optical properties of pure (*34*) and already modified SWCNTs (*34*, *35*). Recently, Heppe *et al.* investigated the solvent isotope effects between H_2_O and D_2_O when exposing SWCNTs to aryl-based diazonium salts (*36*). The reaction of SWCNTs with aryl diazonium salts exhibited strong solvent dependency, suggesting that the surrounding medium considerably affects the process. The deposition of aryl groups onto the SWCNT surface generated a free radical on the SWCNT surface, which was paired with water-derived groups such as ─OH or ─OD. Furthermore, Berger and colleagues exploited the typically used toluene in conjunction with acetonitrile as a co-solvent, which, when used with a potassium acetate (AcOK) as a base and an 18-crown-6 ether as a phase transfer agent, enabled solubilization of a diazonium salt to modify SWCNTs (*24*). Unfortunately, the study of the impact of acetonitrile on the reaction course was beyond the scope of this work. Subsequently, the same group explored the possibility of adding up to 8.3% (each) of a mixture of THF and dimethyl sulfoxide (DMSO) to modify SWCNTs with 2-haloanilines in the presence of potassium tert-butoxide (*t*-BuOK) base (*23*). They concluded that THF speeds up the rate of the reaction by facilitating solubilization of the base in toluene. Additionally, it was deduced that DMSO enhances the basicity of *t*-BuOK, which increases the reactivity of the system. Hence, it is clear that the solvent environment may affect the course of SWCNT modification, but the origins of this phenomenon remain vague.

Concomitantly, one also needs to take into account the presence of the dispersing agent on the SWCNTs, which by themselves cannot be solubilized in water or typical organic solvents (*32*). However, its possible role in the SWCNT functionalization has not been considered before to a sufficient extent. Typically, the consensus is that excess dispersant needs to be removed as it physically hampers SWCNT functionalization by restricting access to its surface (*32*), complicates assembly of SWCNT-based devices (*15*), or impedes charge transport within SWCNT networks (*37*). Still, this step is not considered necessary for SWCNT functionalization when the dispersant concentration is low (*23*) or when it cannot encapsulate SWCNTs tightly (*32*). To alleviate the aforementioned problems, Luo and co-workers performed direct functionalization of bulk unsorted SWCNT powders with diazonium salts generated in situ from the corresponding anilines in acidified water (*38*). This method produced the same photoluminescence (PL) features in SWCNTs as if the material were first suspended in the liquid medium using a dispersant.

In this work, we disentangle the complex solvent-dispersant-SWCNT relationships, which are at the heart of SWCNT functionalization. To reach this goal, we capitalize on the benefits provided by phenylhydrazine (PhN_2_H_3_) derivatives, which, as we recently reported, can be highly versatile agents for SWCNT functionalization in both water and toluene (*39*). Here, we systematically used a broad spectrum of solvents and process conditions, both of which were found to dramatically affect not only the kinetics but also the course of the covalent modification of SWCNT. Moreover, our multiscale modeling (using a fresh implementation of hybrid functional within SIESTA) revealed that solvent characteristics determined the structural reorganization of polymer molecules on the SWCNT surface and charge distribution at the polymer-SWCNT interface, both of which were found crucial to SWCNT reactivity. Hence, the results obtained uncovered that the SWCNT modification can also be tuned not only by reactant choice/concentration but through careful selection of the reaction conditions (solvent type, polymer concentration, and humidity). The use of polar solvents enabled the exclusive generation of the most red-shifted *E*_11_^*-^ peak, which was registered at ∼1300 nm. Last, the high utility of the covalently modified SWCNTs for photonics, prepared according to the described strategy, was demonstrated by illustrating how such materials can serve as proof-of-concept optical nanosensors for cholesterol.

## RESULTS

### Functionalization of dispersant-free SWCNTs

Despite the recent surge of attention devoted to SWCNT functionalization to introduce luminescent defects into the material (*3*, *4*, *15*, *16*), the mechanism of the process remains elusive. To overcome this problem, it is essential to comprehend the interactions between all the entities present in the system (SWCNTs, dispersing agent used to suspend them, solvent environment, and reactive species introduced to be attached to SWCNTs). The influence of the solvent medium and dispersant on SWCNT functionalization appears to be underappreciated, which motivated us to analyze these aspects.

**Fig. 1.**
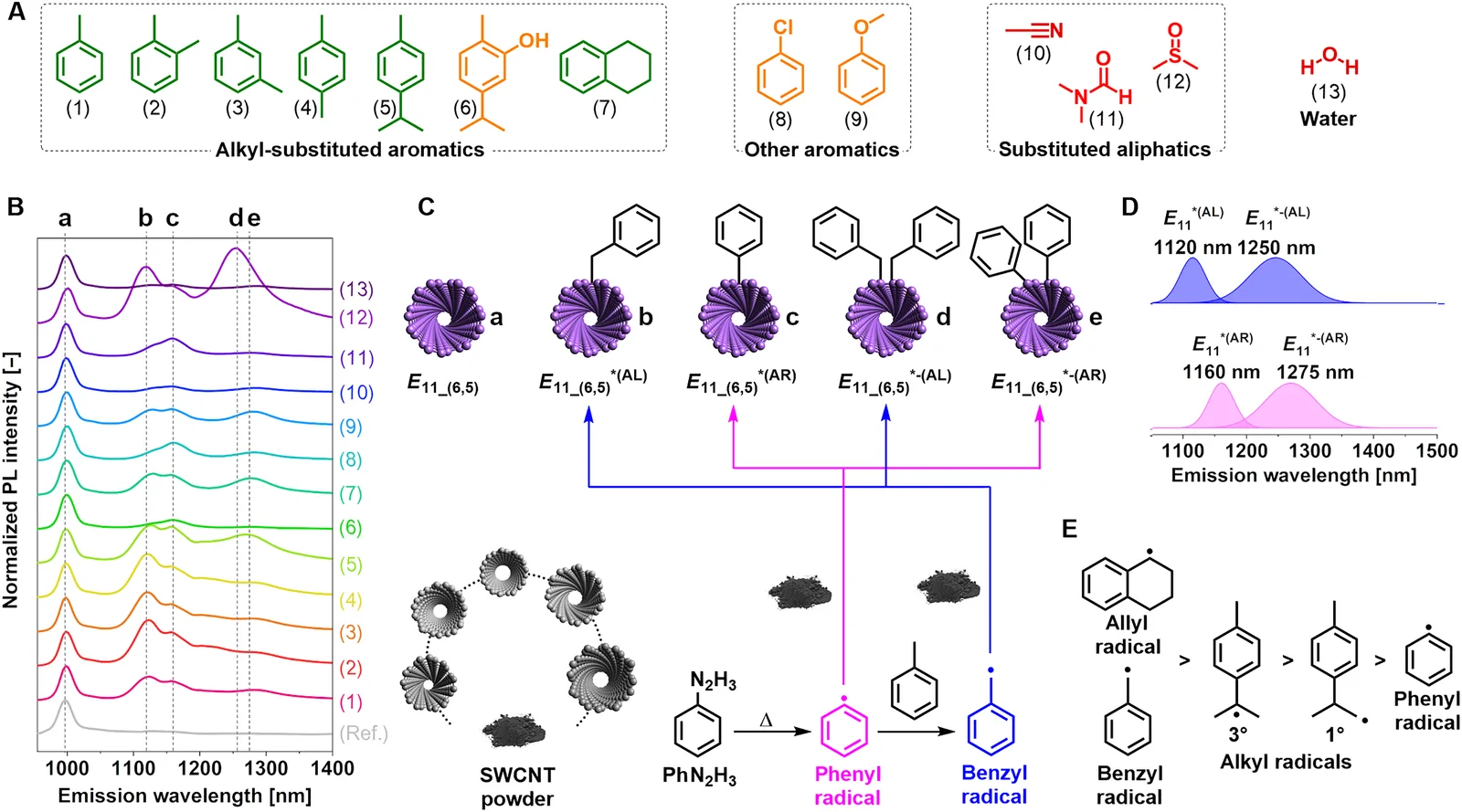
Chemical modification of polymer-free SWCNTs. (**A**) Various solvents used for chemical modification of SWCNTs with PhN_2_H_3_ divided into classes of compounds: (1) toluene, (2) *o*-xylene, (3) *m*-xylene, (4) *p*-xylene, (5) *p*-cymene, (6) carvacrol, (7) tetralin, (8) chlorobenzene, (9) anisole, (10) acetonitrile, (11) DMF, (12) DMSO, and (13) water. Nonpolar, semipolar, and polar compounds are indicated in green, orange, and red, respectively. (**B**) PL spectra (λ_ex_ = 579 nm, solvent: toluene) of raw SWCNT powder functionalized with PhN_2_H_3_ in the solvents, the structures of which were drawn in (A) (see table S1 for estimated peak positions). (**C**) Likely individual optical features (a to e) generated and observed in the PL spectra shown in (B) according to the indicated chemical reactions made possible by the treatment of polychiral SWCNTs with PhN_2_H_3_ in selected solvents (for the sake of clarity, radical transfer to solvent is only presented for toluene), which were subsequently extracted with PFO-BPy6,6′ to isolate modified (6,5) SWCNTs. (**D**) Peaks observed for (6,5) SWCNTs grafted with alkyl (AL) and aryl (AR) moieties (low functionalization degree, left peak; high functionalization degree, right peak). The assignment is based on typical positions of alkyl- and aryl-functionalized SWCNTs (*4*, *24*, *118*), considering solvatochromic shift for samples measured in toluene, compared to water (*35*), and changes to the dielectric environment caused by the presence of polymer rather than surfactant molecules (*119*). (**E**) Most abundant radical species generated while functionalizing SWCNTs in a spectrum of solvents arranged in order of their stability.

First, we investigated the impact of the absence of the polymer on the functionalization of SWCNTs by performing direct chemical modification of raw dispersant-free SWCNT powder (*38*) in various solvents (Fig. 1A, solvent names are provided in the caption). Then, the functionalized SWCNTs were selectively solubilized using PFO-BPy6,6′ to track the extent of chemical modification of only (6,5) SWCNTs as the model material (Fig. 1B), greatly reducing spectral complexity. This step was also necessary as non-individualized SWCNT bundles tend to precipitate in almost any medium. In each case, optical characterization was performed in toluene to eliminate solvent effects that could affect the shape of the polymer corona and the optical readout. Moreover, we assumed that the inherent selectivity of PFO-BPy6,6′ would be preserved, neglecting the possibility that the polymer may preferentially suspend a subset of (6,5) SWCNTs modified in a specific manner.

The spectrum of solvents examined may be divided into three groups: exclusively aromatic (benzene), alkyl-substituted aromatics, other aromatics, substituted aliphatics, and water. The application of alkyl-aromatics (toluene, xylenes, and *p*-cymene) resulted in the formation of distinct peaks in the optical absorption spectra (Fig. 1B). In all these cases, the *E*_11_^*^/*E*_11_ ratio exceeded unity and the PL intensity peaked at ∼1120 nm, which corresponds to the relatively uncommon case of SWCNTs functionalized with alkyl groups [*E*_11_(6,5)_^*(AL)^; Fig. 1, C and D] (*40*). It seems that in the absence of polymer, the generation of the benzyl radical from phenyl radicals produced by the decomposition of PhN_2_H_3_ was preferred. Benzyl radicals are extremely stable species, as they may be stabilized by multiple resonance structures, which increases the likelihood of SWCNT functionalization.

Likewise, the exposure of PhN_2_H_3_ to SWCNTs in tetralin (aliphatic-aromatic compound) also produced an analogous peak, most probably due to the attachment of tetralin molecules to SWCNTs. Tetralin, upon exposure to phenyl radicals from PhN_2_H_3_, may produce allyl radicals (*41*), which also exhibit remarkable stability (Fig. 1E). In turn, these reactive centers may be the point of attachment to the SWCNT surface. In this case, the functionalization of SWCNTs with PhN_2_H_3_ was so effective that a relatively strong peak at ∼1250 nm was detected, which resembles proximal modification (*15*) of the SWCNT surface with alkyl groups [*E*_11_(6,5)_^*-(AL)^; Fig. 1D]. Moreover, both *p*-cymene and carvacrol contain an *i*-propyl group, which, after hydrogen abstraction, gives a tertiary radical, which is renowned for its stability as well, so it should give rise to improved chemical modification of SWCNTs in such media (Fig. 1D). However, only in the former case were the *E*_11_(6,5)_^*(AL)^ and *E*_11_(6,5)_^*-(AL)^ features registered, while the latter medium hindered the modification of SWCNTs altogether. We presume that the inhibition of the SWCNT functionalization in carvacrol stems from the presence of a hydroxyl group in this compound, which is also found in many common radical scavengers (*42*). This hypothesis is supported by the fact that the reaction in structurally similar anisole, for which the hydroxyl moiety (─OH) is protected by a methyl group (─OCH_3_), proceeded to a similar extent as in the case of many other alkyl-substituted aromatics. The same reasoning explains why PhN_2_H_3_ was unable to modify SWCNTs in water.

Chlorobenzene was also evaluated as the solvent to verify our reasoning that benzyl radicals are made in the reaction of Ph• with the above-specified solvents, which may explain the formation of the peak at ∼1120 nm. Because this peak was not detected in the case of chlorobenzene, which lacks alkyl groups, this hypothesis was validated. From the quantitative standpoint, functionalization of SWCNTs in chlorobenzene was modest. A peak centered at ∼1160 nm was observed, corresponding to the attachment of aryl groups to the SWCNT surface [*E*_11_(6,5)_^*(AR)^] as shown in Fig. 1C (*14*, *39*, *40*, *43*, *44*). Phenyl radicals generated by the decomposition of PhN_2_H_3_ are relatively unstable (Fig. 1E). They cannot be stabilized by resonance because the radical orbital is aligned perpendicularly to the π-electron system. Because radical transfer from Ph• to solvent (chlorobenzene) would produce essentially the same reactive species, the extent of SWCNT modification (gauged by the intensity of the *E*_11_^*^ peak to *E*_11_ representing the unmodified SWCNTs) was not substantial.

Last, we also tested three non-aromatic solvents often used in organic chemistry. In acetonitrile, the reaction did not progress, and the SWCNTs remained virtually untouched. Litwinienko *et al.* showed that the reaction rates of specific radical transformations are lower in acetonitrile and propionitrile (*45*) than when conducted in other exclusively hydrocarbon-based media, such as alkanes and benzene. Possibly, the reaction time or temperature was non-optimal for performing SWCNT functionalization in acetonitrile. On the other hand, the reaction conditions were sufficiently suitable for *N*,*N*′-dimethylformamide (DMF), in which aryl groups were attached to the SWCNT surface, which was manifested by an *E*_11_(6,5)_^*(AR)^ peak at ∼1160 nm. It should be noted that this feature is evident for many of the discussed solvents, meaning that the phenyl radicals initially generated from PhN_2_H_3_ were successfully attached to the SWCNT surface, as a considerable amount of them were neither subjected to recombination nor participated in radical transfer. Last, the processing of SWCNTs with PhN_2_H_3_ in DMSO yielded interesting results. Peaks at ∼1120 nm (*E*_11_^*^/E_11_ ≈ 1.65) and 1250 nm (*E*_11_^*-^/*E*_11_ ≈ 2.2) were detected, which align well with the previously discussed *E*_11_(6,5)_^*(AL)^ and *E*_11_(6,5)_^*-(AL)^ alkyl luminescent defects. However, the only alkyl groups in this compound system are in the solvent molecules, suggesting that the procedure may have resulted in the attachment of DMSO molecules to the SWCNT surface. Regardless of the chemical identity of the attached functional group, it is evident that SWCNT functionalization with PhN_2_H_3_ was most pronounced in DMSO. The intensities of the defect-induced *E*_11_(6,5)_^*(AL)^ and *E*_11_(6,5)_^*-(AL)^ peaks were the highest among all solvents examined. Notably, the intensity of the latter exceeded the intensity of the native *E*_11_ transition corresponding to fluorescence from unmodified parts of the SWCNTs. Characterization of the samples by Raman spectroscopy confirms that the highest *I*_D_/*I*_G_ ratio, a measure of the degree of SWCNT functionalization (*46*), was obtained for SWCNTs modified in DMSO (fig. S1A), the most polar solvent used (fig. S1, B and C). Considering all reaction media used, DMSO is perhaps the strongest Lewis base. The Gutmann donor number (DN), which gauges Lewis acid or base strength, places DMSO (29.8) nearly on the same level as aniline (35.0) (*47*). Concomitantly, we have recently demonstrated that PhN_2_H_3_ is most active toward SWCNT functionalization at a basic pH (*39*). While the pH of DMSO cannot be determined, the basic conditions provided by the combination of DMSO (*23*) and PhN_2_H_3_ justify the effectiveness of SWCNT modification under these conditions. From a practical standpoint, it is also important to mention that SWCNT functionalization in various solvents is scalable (fig. S2).

### Functionalization of polymer-wrapped SWCNTs

**Fig. 2.**
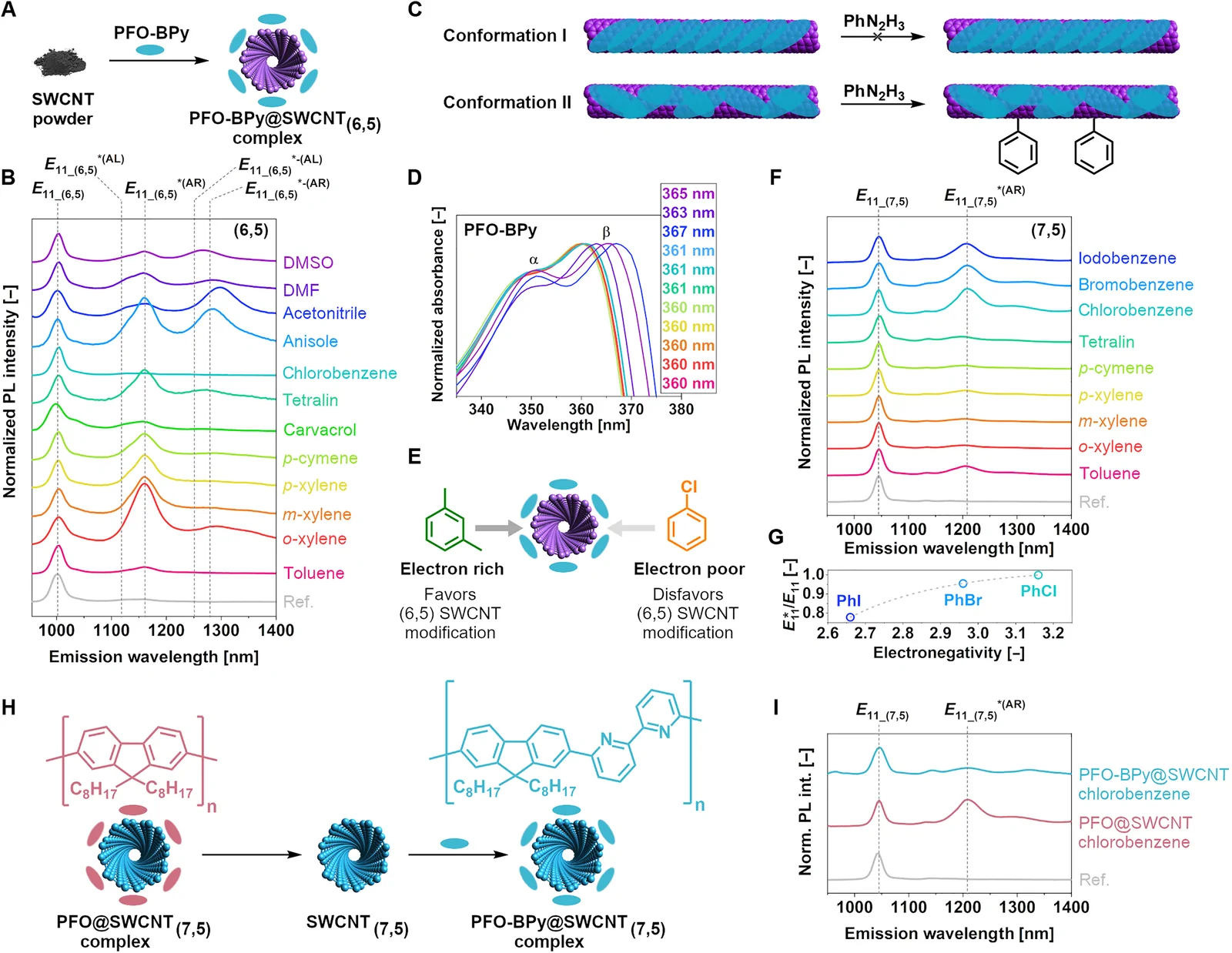
Chemical modification of polymer-wrapped SWCNTs. (**A**) Selective extraction of (6,5) SWCNTs with PFO-BPy giving rise to the formation of the PFO-BPy@SWCNT complex. (**B**) PL spectra (λ_ex_ = 579 nm, solvent: toluene) of (6,5) SWCNTs wrapped with PFO-BPy functionalized in the indicated solvents with PhN_2_H_3_ (see table S2 for estimated peak positions). (**C**) Schematic arrangement of polymer molecules on SWCNTs in two solvents leading to chemical modification of the material and the lack thereof, respectively, depending on the assumed conformation. (**D**) Optical absorption spectra of PFO-BPy registered directly in the examined solvents [color-coded in (B)] along with the positions of absorption maxima of the β phase. (**E**) Improved reactivity of (6,5) SWCNT encapsulated by PFO-BPy in electron-rich solvents. (**F**) PL spectra (λ_ex_ = 642 nm, solvent: toluene) of (7,5) SWCNTs wrapped with PFO functionalized in the indicated solvents with PhN_2_H_3_ (see table S3 for estimated peak positions). (**G**) The relationship between *E*_11_^*^/*E*_11_ ratio of the covalently modified (7,5) SWCNTs and the electronegativity of the halogen atom attached to the benzene ring. (**H**) Replacement of the polymer wrapping on (7,5) SWCNTs from PFO to PFO-BPy. (**I**) PL spectra (λ_ex_ = 642 nm, solvent: toluene) of (7,5) SWCNTs wrapped with PFO and PFO-BPy functionalized in chlorobenzene with PhN_2_H_3_.

The modification of (6,5) SWCNTs with PhN_2_H_3_ progressed differently in the presence of PFO-BPy present on their surface (Fig. 2A). First, the recorded optical absorption spectra of SWCNTs modified in different solvents but characterized in toluene show that the peaks [*E*_11_(6,5)_^*(AL)^] corresponding to the chemical attachment of the alkyl-bearing aromatic solvents positioned at ∼1120 nm disappeared (Fig. 2B). Hence, contrary to previous reports (*23*–*25*, *31*, *40*, *44*), the polymer molecules residing at the SWCNTs not only hinder SWCNT functionalization, but these macromolecules also can alter the reaction course. As a result, the PL spectra were dominated by the *E*_11_(6,5)_^*(AR)^ peaks located at ∼1160 nm, coming from the direct attachment of the phenyl groups to the SWCNTs from PhN_2_H_3_ degradation. Consequently, the polymer-coated SWNCTs favor functionalization with phenyl groups generated by PhN_2_H_3_ decay, and the effect of radical transfer to solvent is minimized. The highest *E*_11_^*^/*E*_11_ ratio of 2.1 was recorded for *o*-xylene.

Second, for the polar solvents such as anisole (*E*_11_^*-^/*E*_11_ ≈ 1.3), acetonitrile (*E*_11_^*-^/*E*_11_ ≈ 1.1), DMF (*E*_11_^*-^/*E*_11_ ≈ 0.4), and DMSO (*E*_11_^*-^/*E*_11_ ≈ 0.4), *E*_11_(6,5)_^*-^ peaks beyond 1200 nm were apparent, indicating a substantial modification of the SWCNT surface, which suggests that these SWCNT solvents favor SWCNT functionalization. We infer that this effect is due to the fact that these liquid media have a different capacity for solubilizing PFO-BPy, which, especially for large molecular weight fractions, suffers from solubility issues (*48*). Consequently, the polymer chains assume different conformation in polar solvents, which leads to pronounced functionalization of SWCNTs (Fig. 2C).

This reasoning is supported by the results of optical characterization of the examined polymers in various solvents (Fig. 2D). For the polar solvents analyzed, the PFO-BPy peak exhibits improved spectral separation, wherein the optical features related to α- and β-phase (*49*–*52*) of the polymer are better resolved. The relative change of intensity of these peaks and dissimilarities in optical absorption wavelengths support the possibility of polymer rearrangement. This phenomenon will be analyzed in greater detail with modeling in subsequent parts of this work. Third, comparison of how the alkyl-containing aromatic solvents affected the extent of SWCNT modification shows that the electron-rich xylenes and analogous alkyl derivatives of benzene performed better than toluene or chlorobenzene, which have a lower electron density. Thus, this observation, combined with the fact that the electron-rich polar solvents described above performed better, suggests that compounds of stronger Lewis base character are better suited for SWCNT modification. This conclusion is congruent with the results of the modification of raw polymer-free SWCNTs explained in the previous section (Fig. 1). In both cases (with and without polymer on the SWCNT surface), relatively electron-deficient toluene and chlorobenzene were least potent for chemical modification of (6,5) SWCNTs, whereas the compounds containing a higher electron density (the polar solvents in particular) promoted SWCNT modification to the most notable extent.

Unexpectedly, the very same solvents (toluene and chlorobenzene) facilitated the chemical modification of the slightly larger (7,5) SWCNTs very well, which were selectively suspended with PFO (Fig. 2F). (7,5) SWCNTs are notoriously hard to modify compared to the most commonly tailored (6,5) SWCNTs (*3*) as their increased diameter reduces the reactivity of the material due to lower curvature (*53*). These unfavorable circumstances are further exacerbated by the fact that, to maintain the stability of the SWCNT dispersions, the thickness of the PFO coating surrounding (7,5) SWCNTs is much larger compared to the (6,5) PFO-BPy wrapping of (6,5) SWCNTs (*48*, *54*). Still, distinct optical peaks at ∼1205 nm related to the modification of SWCNTs of this chirality with aryl groups [*E*_11_(7,5)_^*(AR)^] were registered in the selected solvents. Based on this outcome, it can be concluded that the PFO@ (7,5) SWCNT system exhibits a contrasting behavior to the PFO-BPy/ (6,5) SWCNT one. Previously, we demonstrated that conjugated polymer/SWCNT complexes respond differently to solvents depending on their modality (*55*). In brief, the molecules of conjugated polymers on the SWCNT surface exert strain on them, and the strain component is positive for mod = 1 SWCNTs, while it is negative for mod = 2 ones. Hence, we hypothesize that these contrasting characters of (6,5) (mod = 1) and (7,5) (mod = 2) SWCNTs, combined with the different behavior of the PFO-BPy and PFO, respectively, encapsulating them, may justify the observed effect. This justification is further supported by the results of Capaz *et al.* (*56*) and Kim *et al.* (*57*), who reported how SWCNTs of dissimilar modality behave differently.

To prove that the electron-poor solvents favor modification of (7,5) SWCNTs are not isolated incidents, (7,5) SWCNTs were exposed to PhN_2_H_3_ in a selection of halogen-containing aromatic solvents (chlorobenzene, bromobenzene, and iodobenzene). The more electron-deficient the system was, gauged by the electronegativity of the attached halogen atom, the more it favored (7,5) SWCNT modification (Fig. 2G). The highest *E*_11_^*^/*E*_11_ ratio was recorded in chlorobenzene (*E*_11_^*^/*E*_11_ ≈ 1.0), for which the electronegativity of the chlorine atom was 3.16, compared to 2.96 and 2.66 for bromine (*E*_11_^*^/*E*_11_ ≈ 0.9) and iodine (*E*_11_^*^/*E*_11_ ≈ 0.7), respectively (*58*–*61*). The *I*_D_*/I*_G_ ratio recorded by Raman spectroscopy, which gauge the level of structural imperfection in SWCNTs, was also the highest for (7,5) SWCNTs processed in chlorobenzene (fig. S3).

Notably, the reactivity of (7,5) SWCNTs can also be controlled by the character of the conjugated polymer encapsulating them. The replacement of PFO, which was used for selective isolation of (7,5) SWCNTs with PFO-BPy (Fig. 2H), strongly decreased the reactivity of (7,5) SWCNTs (Fig. 2I). The *E*_11_^*^/*E*_11_ ratios of (7,5) SWCNTs coated with PFO and PFO-BPy, which were functionalized with PhN_2_H_3_ in chlorobenzene, reached 1.03 and 0.22, respectively. Pyridine is a Lewis base (*62*), so its presence in PFO-BPy enables the formation of n-π* interactions between the two lone electron pairs located at the nitrogen atoms in the polymer and the sp^2^ carbon lattice of SWCNTs. The results of other experiments described above show that when (7,5) SWCNTs are in an electron-rich environment, their reactivity is moderated, which explains why the PFO-BPy@ (7,5) SWCNT complex was much less prone to chemical modification than PFO@ (7,5) SWCNT.

**Fig. 3.**
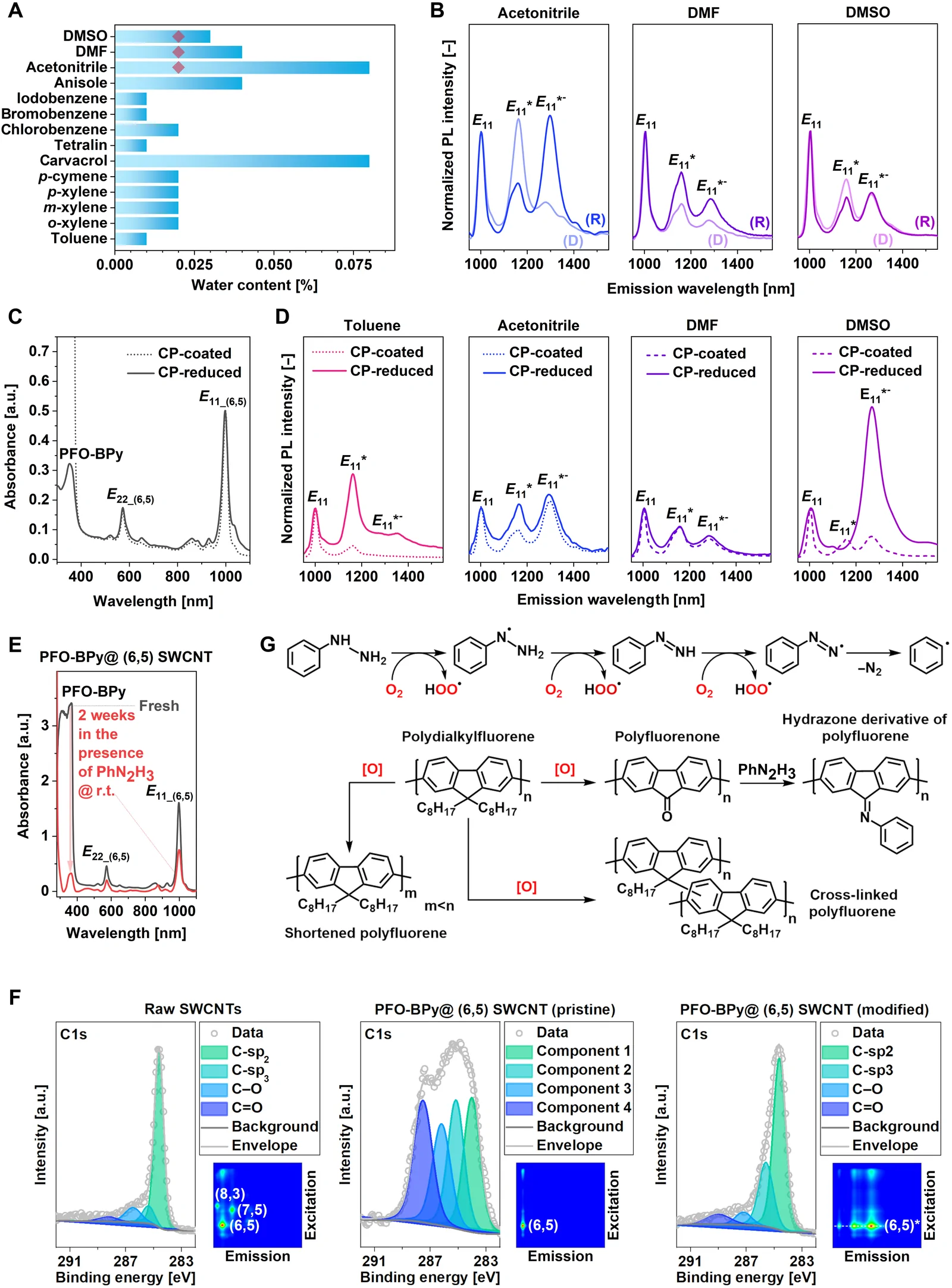
The impact of polymer and water on the propensity of SWCNTs for modification. (**A**) Moisture content in the solvents used for SWCNT functionalization [the content of water in selected solvents dried with molecular sieves examined in more detail in (B) is indicated with red rhombi]. (**B**) PL spectra (λ_ex_ = 579 nm, solvent: toluene) of (6,5) SWCNTs wrapped with PFO-BPy functionalized in the indicated solvents with PhN_2_H_3_ before (R, regular) and after drying (D, dried) (see table S4 for estimated peak positions). (**C**) Optical absorption spectra of (6,5) SWCNTs suspended with PFO-BPy in toluene before and after purifying from the polymer. (**D**) PL spectra (λ_ex_ = 579 nm, solvent: toluene) of (6,5) SWCNTs wrapped with PFO-BPy functionalized in the indicated solvents with PhN_2_H_3_ before and after removal of the conjugated polymer (CP) from the SWCNT surface (see table S5 for estimated peak positions). (**E**) Optical absorption spectra of as-made PFO-BPy@ (6,5) SWCNT suspension and after exposure to PhN_2_H_3_ for 2 weeks at room temperature (r.t.). (**F**) C1s spectra recorded by XPS of the raw SWNCT powder, SWCNT suspension prepared by selective wrapping with PFO-BPy (components not defined), and PFO-BPy@ (6,5) SWCNT subjected to functionalization with PhN_2_H_3_ (1 μl/ml) in DMSO at 80°C for 2 hours. Insets show corresponding PL excitation-emission maps. (**G**) Reactions generating phenyl radicals from PhN_2_H_3_ and possible transformations experienced by the polyfluorene-based polymers and copolymers. Pyridine units are not shown for the sake of clarity. a.u., arbitrary units.

Apart from the chemical identity of the solvent or the polymer wrapping, radical reactions are susceptible to the presence of moisture (*40*). Hence, we measured the water content of the examined solvents before and after drying them with 4 Å molecular sieves by means of Karl Fischer titration (Fig. 3A). In most cases, the moisture level was relatively low and close to the detection limit of the used instrument. The highest water content was recorded for carvacrol and the polar solvents used. The former compound contains a hydroxyl group, which increases its hydrophilicity. The latter compounds, on the other hand, due to their chemical character, can readily interact with water. As a matter of fact, they are fully miscible with water (*63*), which should give rise to homogeneous distribution of water in these liquid media (no phase separation).

The reactivity of PhN_2_H_3_ in the polar liquid media changed to a considerable extent depending on whether water was present or removed (Fig. 3B). The most apparent difference was the increased preference for the formation of the *E*_11_^*^ peak after drying, corresponding to a modest degree of implantation of luminescent defects, rather than *E*_11_^*-^, which is indicative of a substantial density of such defects. While molecular sieves are widely recognized for their ability to selectively adsorb moisture, their additional impact on the chemical environment has often been overlooked. They not only remove water but also affect the acidity of polar solvents (*63*, *64*), a phenomenon that critically affects our reaction pathways. Additionally, such sieves often also contain acidic or basic sites and can be used as acid/base catalysts (*64*). The molecular sieves used in this work are composed of sodium aluminosilicate, which acts as a Lewis acid, altering the proton availability in certain solvents.

We previously concluded that the Lewis base character of the solvent enhances the reactivity of SWCNT-based systems and promotes a higher degree of chemical modification, often resulting in the emergence of an *E*_11_^*-^ peak beyond 1250 nm. Hence, the acidic character of the molecular sieves reduces this tendency and better supports the generation of an *E*_11_^*^ peak corresponding to moderately functionalized SWCNTs. The most notable difference, supporting this hypothesis, was observed in the case of acetonitrile, for which the preference for the formation of *E*_11_^*^ and *E*_11_^*-^ peaks flipped upon drying the solvent. The relatively high content of water, which can be considered as a Lewis base, in unprocessed acetonitrile, likely promoted the implantation of a substantial number of luminescent defects, compared to other solvents. It should also be highlighted that the position of the *E*_11_^*-^ peak maximum for acetonitrile reached 1300 nm (Figs. 2B and 3, B and D). Thus, drying solvents using molecular sieves may not merely remove water but also induce crucial changes for the nanochemistry of low-dimensional materials.

Comparing the functionalization of polymer-free and polymer-wrapped SWCNTs, we see that polymer presence on the SWCNT surface may facilitate or hinder the introduction of luminescent defects into SWCNTs, depending on the solvent (Figs. 1B and 2B). At the same time, a complete lack of polymer favors the emergence of multiple *E*_11_^*^ features, especially those that originate from the radical transfer to the solvent [*E*_11_(6,5)_^*(AL)^] at ∼1120 nm. On the contrary, the polymer-wrapped SWCNTs prefer aryl functionalization [*E*_11_(6,5)_^*(AR)^], which manifests at ∼1160 nm. Therefore, we explored how reducing the polymer content in the selectively extracted (6,5) SWCNTs would affect their modification. Seeking the best materials for emission in the further parts of the NIR spectrum, we focused our analysis on SWCNTs processed in polar aprotic solvents, which displayed the most prominent red-shifts (Figs. 1 and 2). Toluene was used as a reference solvent. In the literature, it is generally accepted that minimizing the polymer content improves the degree of SWCNT functionalization (*23*, *39*). However, this knowledge is a generalization resulting from the evaluation of how SWCNTs behave in toluene. It is therefore desired to extend the scope of the liquid media to validate this relatively widespread assumption. The optical absorption spectra demonstrated a successful reduction of the amount of polymer, the intensity of the optical transition of which decreased by two orders of magnitude (Fig. 3C). While the reduction of the PFO-BPy content on the surface of (6,5) SWCNTs did indeed improve the reactivity of SWCNTs in toluene (Fig. 3D), the effect of such processing was more complex for other solvents. In the case of acetonitrile, the relative intensity of *E*_11_^*^ and *E*_11_^*-^ peaks increased (the former optical feature in particular). Acetonitrile may have enlarged the solvent-accessible surface area by rearranging the polymer molecules on the SWCNT surface, thereby reducing the likelihood that two functional groups will attach in the vicinity of one another. Furthermore, the functionalization of (6,5) SWCNTs with reduced polymer content in DMF using PhN_2_H_3_ also improved the functionalization degree, but to a smaller extent. Last, the most beneficial result was obtained for the covalent modification of SWCNTs in DMSO after partial removal of the polymer. Under such conditions, the SWCNTs were substantially modified, and almost exclusive *E*_11_(6,5)_^*-(AR)^ defect emission centered at 1280 nm (with *E*_11_^*^/*E*_11_ ratio of ∼3.1) was noted (see below for theoretical explanation).

Hence, the presence of the polymer strongly influences the modification process. Not only does such polymer coating affect the degree of chemical modification of SWCNTs, but the emergence of different optical spectra also strongly suggests that the polymer molecules may affect the type and concentration of reactive species in the system. The polymer itself is not resistant to the reactive species present in the medium and displays a self-cleaning capacity. Such a feature has not been reported for other SWCNT modification strategies, making this functionalization platform unique. Removal of the polymer from the SWCNT surface is highly challenging (*65*), which results in considerable material loss. Hence, efforts have been made to design depolymerizable dispersants (*66*–*68*). Unfortunately, such conjugated polymers do not exhibit similarly impressive SWCNT extraction selectivity as the well-known PFO-BPy (*69*), PFO-BT (*53*), or PFO (*70*). The recorded optical absorption spectra show that upon leaving the PFO-BPy@ (6,5) SWCNT sample exposed to PhN_2_H_3_ for a prolonged period, the intensity of the peak corresponding to the polymer was dramatically reduced (Fig. 3E). To validate that the treatment of SWCNTs with PhN_2_H_3_ may favorably disturb the polymer corona around SWCNTs, we examined the material by x-ray photoelectron spectroscopy (XPS) (Fig. 3F). The raw SWCNT powder was primarily made of sp^2^-hybridized carbon atoms, judging by the sharp nature of the C1s peak. Upon coating of the material with PFO-BPy, considerable C1s peak broadening was observed. The data resembled a typical spectrum of a conjugated polymer (*71*), which matched the expectations since PFO-BPy creates a dense layer around SWCNTs (*48*). Last, upon treating the PFO-BPy@ (6,5) SWCNT material with PhN_2_H_3_, the resulting C1s peak was not broad. Compared with the spectrum registered for untreated SWCNTs, we noted an increased content of sp^2^-hybridized carbon caused by addition of luminescent defects. Hence, we proved that exposing SWCNTs to PhN_2_H_3_ alleviated the issue of excessive unwanted polymer molecules on SWCNTs.

Several chemical transformations may be responsible for this effect (Fig. 3G). First, the conversion of PhN_2_H_3_ to phenyl radicals generates hydroperoxyl radicals (HOO•) (*39*), which are potent oxidants (*72*). In the presence of these and other reactive species, as well as oxygen dissolved in toluene, the polyfluorene framework may be subject to main chain scission. The increased length of the side chain promotes such a breakup, so polymers made of dioctylfluorene units may undergo this undesired transformation, reducing their molecular weight (*73*) and increasing the probability of their detachment from the SWCNT surface (*48*). Second, a common problem associated with the application of polyfluorene compounds is caused by their tendency to undergo oxidation to a corresponding ketone, i.e., polyfluorenone (*74*–*77*). This degradation mechanism, which hinders the use of polyfluorene-based light-emitting devices, is likely to occur under the above-explained oxidizing conditions. As shown in the cited literature, this issue is also persistent in dialkyl-substituted fluorenes. In the case described here, the situation is more complex as PhN_2_H_3_ may react with the as-formed carbonyl group of the polyfluorenone to produce a corresponding hydrazone derivative (*78*). Third, in the presence of oxygen and reagents of similar nature, polyfluorene may exhibit cross-linking (*79*, *80*). Considering that the above-described results show that uncovering the SWCNT surface increases the reactivity of SWCNTs, the capacity of the PhN_2_H_3_-driven functionalization system to perform polymer rearrangement autonomously is highly beneficial.

### Multiscale modeling of SWCNT-polymer-solvent interactions

**Fig. 4.**
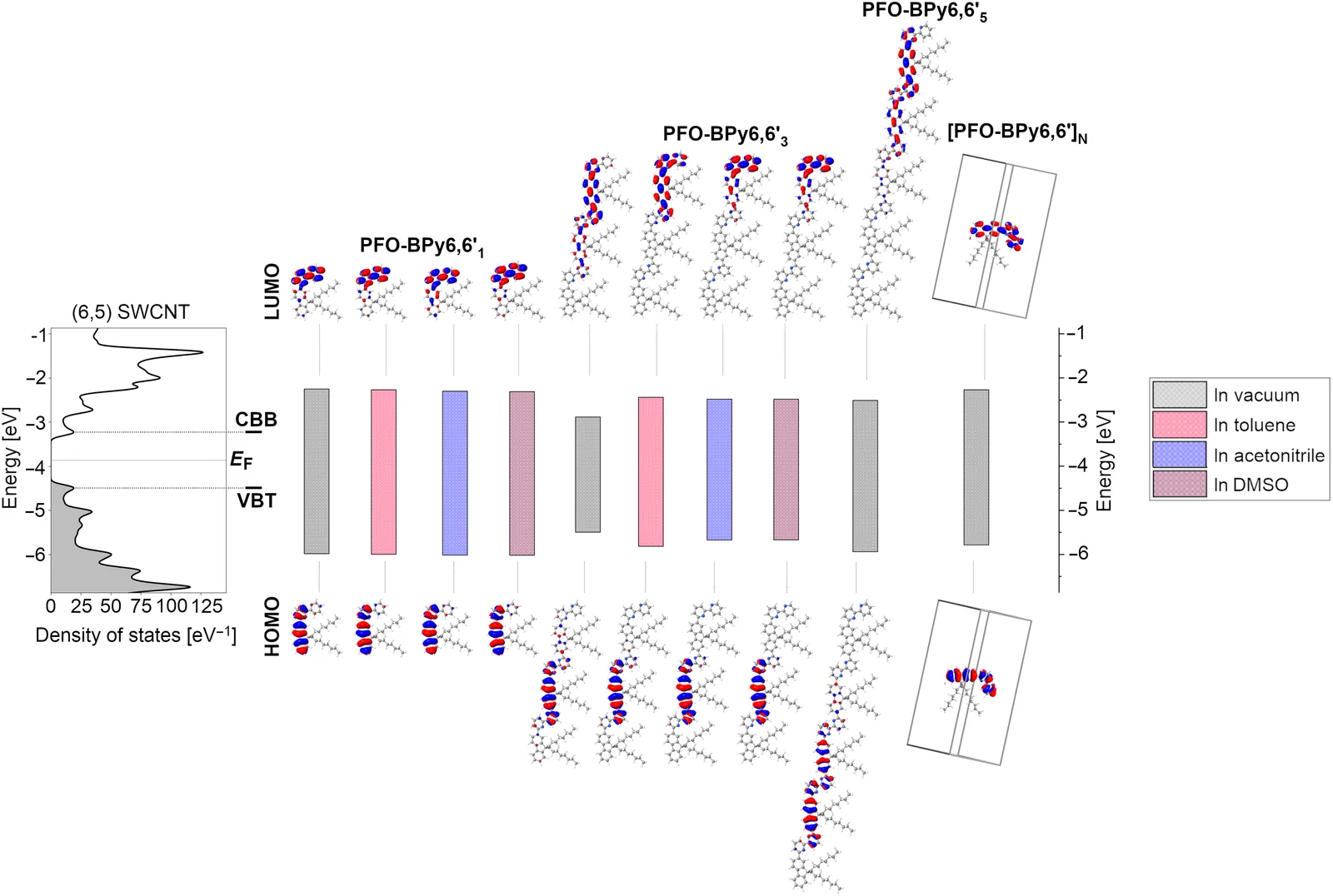
PDOS of (6,5) SWCNT and energy levels of PFO-BPy6,6′ polymer of varying lengths. The results were obtained using SP-DFT with the HSE06 hybrid functional, PseudoDojo pseudopotentials, and a medium basis set. Polymer energy levels are aligned to the Fermi level (*E*_F_) of the SWCNT and presented in absolute energy units. The valence band top (VBT) and conduction band bottom (CBB) of the SWCNT are indicated. HOMO and LUMO levels of fully relaxed polymers are depicted as vertical bars, calculated in four different environments: vacuum (gray), toluene (pink), acetonitrile (navy), and DMSO (purple). The spatial distributions of the HOMO and LUMO orbitals are visualized at an isovalue of 0.06 Å^−3/2^, with red and blue lobes representing the negative and positive regions of the wave function, respectively. On the far right, the orbital distribution for the infinite polymer is shown within a simulation cell. Carbon, nitrogen, and hydrogen atoms are colored gray, blue, and white, respectively.

To gain deep insight into the impact of various solvents on the interaction between polymers and (6,5) SWCNTs, we used spin-polarized density functional theory (SP-DFT) (*81*, *82*), incorporating solvent effects via the COSMO continuum solvation model (*83*, *84*), as well as molecular dynamics (MD), time-stamped force-bias Monte Carlo (TFMC) (*85*, *86*), and density functional–based tight-binding (DFTB) modeling (*87*) (see the Supplementary Materials for details). Given that charge transfer between a conjugated polymer and a specific SWCNT type may influence chirality-selective extraction (*53*, *88*), we analyzed the energy level diagram, aligning the calculated highest occupied molecular orbital (HOMO) and lowest unoccupied molecular orbital (LUMO) levels of monomeric, trimeric, pentameric, and infinite-chain PFO-BPy6,6′ polymers to the projected density of states (PDOS) of the SWCNT (Fig. 4). To ensure the robustness of these calculations, we compared results obtained using a newly implemented HSE06 exchange-correlation functional (*89*, *90*) in SIESTA (*91*, *92*) with those from QuantumATK (*93*, *94*) (fig. S4). HOMO and LUMO spatial distributions are consistent across both approaches, confirming that the observed electronic trends are independent of basis set and code-specific implementations. To assess the impact of solvent environments on the polymer’s frontier orbitals, we performed calculations both in vacuum and using the COSMO model to simulate toluene, acetonitrile, and DMSO environments.

For the monomer, HOMO and LUMO energy levels remained essentially congruous across different environments. However, for the trimer, the presence of solvent led to a stabilization of the HOMO levels (more negative values) and a destabilization of the LUMO levels (less negative values), effectively narrowing the HOMO-LUMO gap. This trend aligns with literature reports indicating that solvent polarity can influence the electronic properties of conjugated systems (*95*). Regarding orbital shapes, HOMOs were relatively delocalized across the polymer backbone in all environments. In contrast, LUMOs exhibited increased localization on the bipyridine (BPy) units with chain elongation, a phenomenon that was more pronounced in polar solvents such as acetonitrile and DMSO. This enhanced localization suggests stronger electron-accepting behavior of the BPy units in polar environments. For the infinite polymer chain, calculations performed in vacuum revealed that both the HOMO and LUMO orbitals were delocalized along the polymer backbone, indicating a uniform electronic distribution. The energy level alignment suggests that the polymer’s HOMO lies below the SWCNT valence band edge, and its LUMO lies above the conduction band edge. This configuration corresponds to a broken-gap or type-Ib heterojunction, suggesting that direct charge transfer under equilibrium conditions may be energetically unfavorable. However, photoinduced or field-assisted processes could still enable charge separation, depending on the environment and polymer length.

Closer inspection of polymer morphologies reveals substantial solvent-induced structural reorganization in PFO-BPy6,6′ polymers, highlighting that vacuum-optimized geometries do not adequately capture conformational behavior under realistic conditions. The extent of these structural changes correlates with solvent polarity and is most pronounced in polar environments such as acetonitrile and DMSO, where deviations from vacuum structures are the greatest (fig. S5). Overall, structural reorganization is more notable for the trimer than for the monomer in any given solvent, likely due to the increased conformational flexibility and the larger number of solvent-sensitive sites along the extended backbone and side chains. As expected, the most pronounced deviations occur at the polymer termini, involving not only the BPy units but also the alkyl side chains, suggesting that both the conjugated core and peripheral groups respond to the surrounding dielectric environment.

These solvent-induced structural reorganizations are accompanied by pronounced changes in solvation energetics (fig. S6). While the spatial distribution of the solvent-accessible surface charge density remains broadly consonant across solvents, the total solvation energy varies markedly with both solvent polarity and polymer length. This result highlights that even subtle geometric rearrangements can lead to substantial differences in electrostatic stabilization. The most negative surface charge densities are mainly located on the alkyl side chains. At the same time, the nitrogen atoms in the conjugated backbone exhibit the most positive regions, indicating that both polar and dispersive interactions play a role in modulating solvation.

**Fig. 5.**
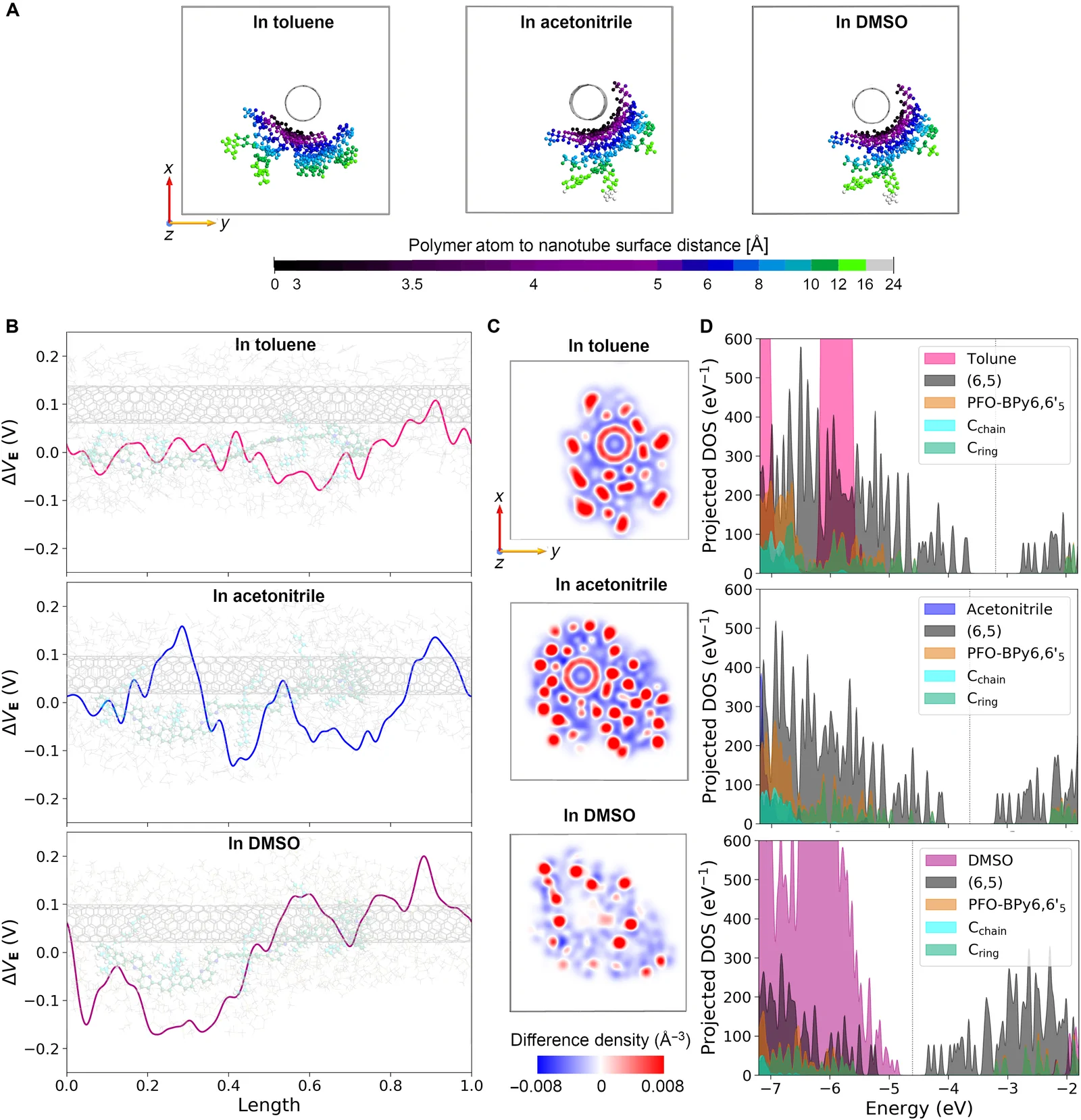
Computed structural and electronic properties of (6,5) SWCNT interacting with PFO-BPy6,6′_5_ polymer in toluene, acetonitrile, or DMSO solutions obtained at the DFTB level. (**A**) Atomistic cross-sectional views of each system. SWCNTs and polymers are shown using stick and ball-and-stick models, respectively. Solvent molecules are omitted for clarity. Polymer atoms are colored based on their distance from the SWCNT lateral surface, with darker shades indicating closer proximity and lighter shades indicating greater distance. (**B**) Electrostatic difference potential (Δ*V*_E_) plotted as a 1D projection on the *z* axis (along SWCNT symmetry axis). Δ*V*_E_ is the difference between the electrostatic potential of the self-consistent valence charge density and the electrostatic potential from a superposition of atomic valence densities. SWCNTs and polymers represented by stick and ball-and-stick models are shown on all cut-planes, while solvent molecules are represented as lines. (**C**) Electron difference density (EDD) maps showing the difference between the self-consistent valence charge density and a superposition of atomic valence densities. The *xy* cut planes are taken at 0.4 (across the SWCNT and polymer complex) of the fractional length of the simulation boxes along the *z* axes. (**D**) The PDOS, shown on an absolute energy scale, is presented for the SWCNT, polymers, solvents, and distinct regions (C_ring_ and C_chain_) of the polymers for both systems. The Fermi level is indicated by a dotted line.

We performed additional DFTB simulations to identify the influence of solvent on polymer-SWCNT interactions. Due to computational limitations, the systems used for DFTB modeling were constructed by reducing the size of those used in the MD/MC simulations shown in fig. S7A. Specifically, smaller subsystems were extracted from the final MD/MC snapshots, preserving an ∼8-Å thick solvent shell around both the SWCNT and the polymer. Cross-sectional views obtained from the DFTB simulations (Fig. 5A) reveal pronounced solvent-dependent differences in how the PFO-BPy6,6′ polymer conforms to the (6,5) SWCNT, highlighting structural features not captured in classical MD/MC simulations. In acetonitrile and DMSO, the polymer wraps more closely around the SWCNT, with a greater number of atoms located in close proximity to the SWCNT surface. At the same time, portions of the polymer, particularly the side chains, extend further away from the SWCNT compared to the toluene case. This suggests that, in polar solvents, the polymer adopts a more spatially extended but closely wrapping conformation. In contrast, in toluene, the polymer appears more condensed overall, yet less intimately wrapped around the SWCNT. These structural differences are further illustrated in fig. S9, where a different viewing angle of the colored-atom visualization shows how polymer atoms are distributed along the SWCNT, helping to identify which regions of the polymer lie closer or further from the SWCNT surface.

These structural variations are accompanied by substantial differences in electrostatic potential (Fig. 5B) and charge distribution (Fig. 5C) across the three solvent systems. A comparison of the electrostatic difference potential (Δ*V*_E_) along the SWCNT interacting with PFO-BPy6,6′ showed a marked contrast between the system in toluene and those in acetonitrile and DMSO. Δ*V*_E_ highlights spatial regions where considerable charge redistribution occurs, relative to the sum of electron densities of neutral, non-interacting atoms (Fig. 5B). Both acetonitrile and DMSO induce much larger variations in Δ*V*_E_ than toluene. In all cases, Δ*V*_E_ oscillates around zero in regions where the SWCNT interacts with the polymer. Immersing the SWCNT-polymer complex in acetonitrile results in more positive Δ*V*_E_ values when the polymer side chains are adjacent to the SWCNT, and more negative values when the polymer backbone is nearby. In contrast, the system in DMSO exhibits an opposite trend: Δ*V*_E_ becomes more negative when the side chains are near the SWCNT and more positive when the backbone is closer.

These solvent-dependent differences suggest that the nature of the polymer-SWCNT interaction is strongly modulated by the surrounding dielectric environment. This effect is also clearly visible in the cross-sectional charge density maps shown in Fig. 5C. In both toluene and acetonitrile, a blue region, indicating electron deficiency, is observed inside the SWCNT, whereas a red region, representing electron accumulation, is localized on the SWCNT wall. This redistribution pattern is absent in DMSO, where the difference density across the SWCNT cross-section is nearly zero. Solvent-dependent variations in electrostatic potential and charge density reveal that polar solvents such as acetonitrile and DMSO notably modulate the electronic environment of the polymer-SWCNT interface. While toluene preserves weak, localized interactions, polar solvents induce stronger or inverted charge redistribution patterns, suggesting solvent-mediated control over polymer-SWCNT coupling.

Solvent-mediated differences in the interaction between the PFO-BPy6,6′ and (6,5) SWCNT are also reflected in the electronic structure of the combined systems. Figure 5D presents the PDOS for the solvent, SWCNT, polymer, and distinct polymer regions (main chain/side chains) in each solvent environment. A shift of the Fermi level toward more negative energies is observed in the polymer-SWCNT complex immersed in acetonitrile, compared to toluene. An even larger shift to lower energies occurs in the presence of DMSO. All states associated with the SWCNT and polymer in the (6,5) + PFO-BPy6,6′ + DMSO system are shifted to lower absolute energy values relative to those in the toluene-based system. Notably, in contrast to the toluene and acetonitrile systems, DMSO-related states appear within the bandgap region, effectively reducing the system’s bandgap and forming a new valence band edge. Additionally, the DMSO states show greater overlap with both SWCNT and polymer states over a broader energy range than is observed for toluene or acetonitrile, suggesting stronger electronic coupling and more pronounced solvent-system hybridization. These effects are consistent with the experimentally observed solvent-dependent functionalization behavior and suggest that solvent-induced changes in the electronic structure of the polymer-SWCNT complex may influence its reactivity.

Last, polar aprotic solvents, such as acetonitrile or DMSO, are unable to provide a good solvation for radicals and anions (*96*). The absence of hydrogen atoms bonded to electronegative elements, such as O or N, prevents them from forming hydrogen bonds that would be able to stabilize these species through the so-called cage effect (*97*). Consequently, because they are more suited to stabilize cations, these solvents enhance the reactivity of nucleophiles in nucleophilic aromatic substitutions like S_N_2 and S_N_Ar and radical species containing heteroatoms (*98*, *99*). This phenomenon has important implications for the reaction systems analyzed here. Wang and co-workers reported that nucleophiles may act as pairing groups and the light emission from the defect site systematically narrows and red-shifts with sterically larger aryl and pairing groups (*29*). While the reaction system studied in the indicated work was not based on radicals, such as the one discussed here, it likely exhibits a similar phenomenon. Using acetonitrile or DMSO as the medium for SWCNT functionalization likely enhances the reactivity of PhN_2_H_3_-derived heteroatom-containing radicals, which mimic the behavior of nuclelophiles, by not being able to stabilize them. This may explain why strongly red-shifted PL features, particularly *E*_11_^*-^ peaks, were evident for such solvents.

### The application of chemically modified SWCNTs for cholesterol sensing

Currently, SWCNT-based optical sensors are widely used for detecting biogenic amines, such as dopamine and serotonin (*7*, *32*). However, this low-dimensional material can sense other vital molecules. Cardiovascular diseases are the leading cause of death globally (*100*). A reduction in cholesterol concentration in the blood could considerably decrease the risk of death due to this condition (*101*), so it would be valuable to have the capacity to track the amount of this compound in a more accessible manner. Recently, SWCNTs enhanced with luminescent defects have revealed that the optical characteristics of such materials are sensitive to cholesterol (*9*). However, with the increase in cholesterol concentration, the luminescence intensity of the SWCNTs generally decreased. This effect reduces the utility of such nanosensors, as the higher the cholesterol concentration, the more challenging it becomes to detect the optical signature of its presence.

**Fig. 6.**
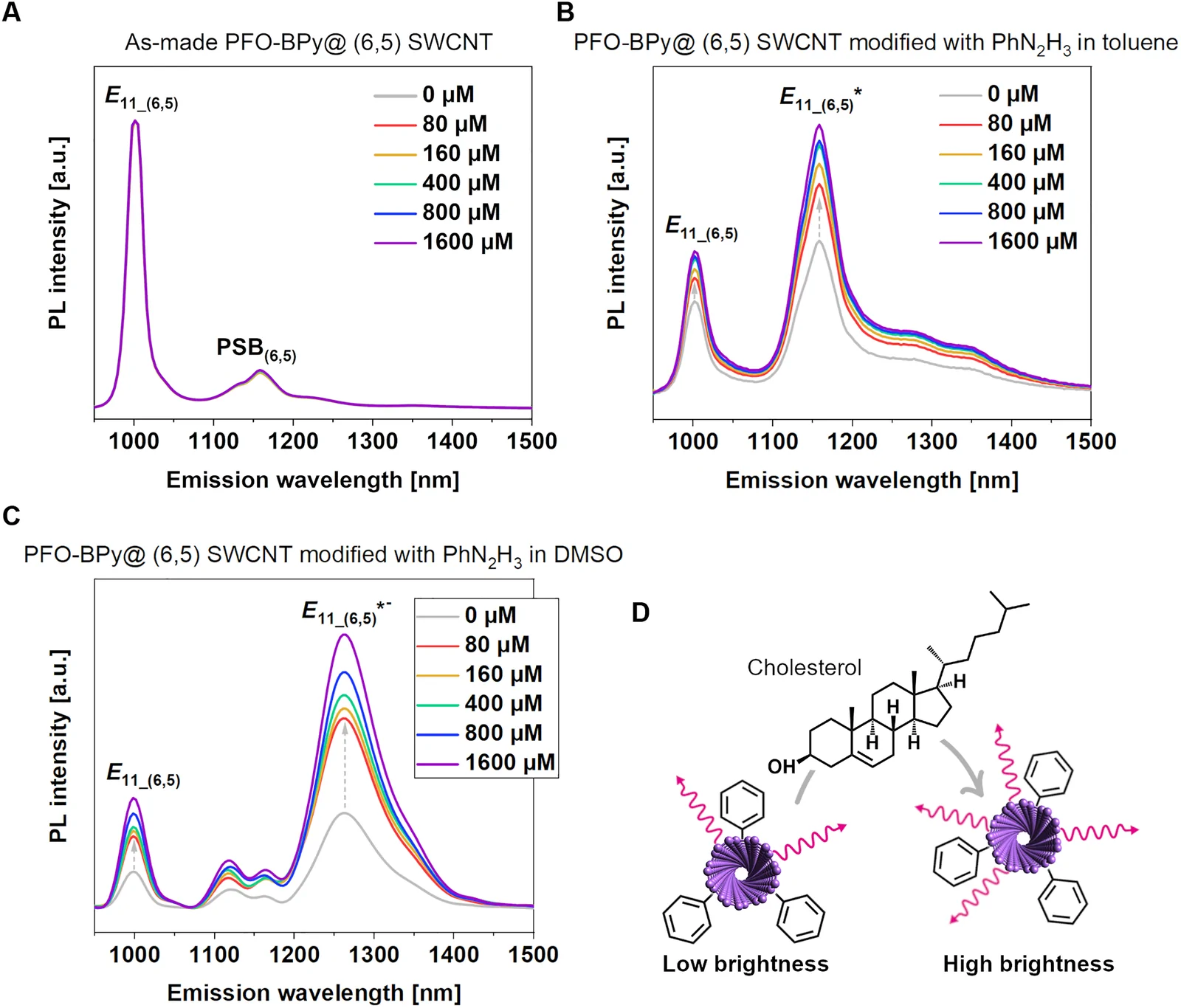
Optical detection of cholesterol with modified SWCNTs. PL spectra (λ_ex_ = 579 nm, solvent: toluene) of (**A**) pristine (6,5) SWCNTs wrapped with PFO-BPy and functionalized with PhN_2_H_3_ in (**B**) toluene, and (**C**) DMSO as a function of concentration of cholesterol, to which the samples were exposed. (**D**) Brightening of SWCNTs modified with luminescent defects in the presence of cholesterol. PSB, photoluminescence sideband (*22*).

Regardless of cholesterol concentration, the PL spectra of pristine post-CPE SWCNTs remained intact, as the absolute intensity of the *E*_11_ peak remained unchanged (Fig. 6A). However, the SWCNTs (covalently modified according to the methodology reported here), which displayed the defect-related *E*_11_^*^ (Fig. 6B) and *E*_11_^*-^ (Fig. 6C) peaks created in toluene and DMSO, respectively, exhibited a different behavior. In both cases, upon exposure to cholesterol, the fluorescence intensity of such SWCNTs increased. A gradual increase in cholesterol concentration resulted in a corresponding brightening of SWCNTs, facilitating cholesterol sensing (Fig. 6D). The relative intensity of the defect-induced *E*_11_^*^ emission increases with cholesterol concentration, making it possible to monitor its concentration in a ratiometric manner (fig. S10). This outcome highlights the high application potential of the materials designed in this study, whose optical characteristics were tuned by selecting an appropriate solvent for functionalization.

## DISCUSSION

The carried-out work sheds considerable insight into the solvent-dispersant-SWCNT interactions, which determine the reactivity of SWCNTs. The inclusion of polymer molecules on the SWCNT surface strongly affects the types of radicals generated and subsequently attached to the SWCNTs, which in turn determine their resulting optical properties. This finding clarifies a common misconception that the dispersant molecules merely block the access of reactive species to SWCNTs. We also found that polar solvents promote high-degree functionalization of SWCNTs, which is manifested by the emergence of red-shifted optical features coinciding with the telecommunication part of the NIR range. The very much improved propensity of SWCNTs for functionalization in polar solvents results from better charge redistribution at the polymer-SWCNT interface occurring in such chemical environments. Hence, this noted relationship provides powerful means to tailor the surface of SWCNTs and other relevant low-dimensional materials. Last, the SWCNTs crafted according to the reported strategy have the capacity to detect cholesterol, and as cholesterol levels increase, their brightness improves, further enhancing their utility.

## MATERIALS AND METHODS

The materials used in the experiments were used in the form in which they were supplied. The purity of the reagents, along with the manufacturer and data allowing for identification, can be found below.

### Raw SWCNT materials

This study was carried out using (6,5)-enriched CoMoCAT SWCNTs (Sigma-Aldrich, product number: 773735, lot: MKCM5514, purity: 95%-carbon basis).

### Reagents for CP synthesis

The following reagents were used: 9,9-dioctylfluorene-2,7-bis(boronic acid pinacol ester) (Angene, catalog no.: AG0034EZ, CAS: 196207-58-6, purity: 98%), 2,7-dibromo-9,9-dioctyl-9*H*-fluorene (Sigma-Aldrich, catalog no.: 560073-25 g, CAS: 198964-46-4, purity: 96%), Aliquat 336 TG (Alfa Aesar, catalog no.: A17247, CAS: 63393-96-4, purity: N/A), and tetrakis(triphenylphosphine)palladium – Pd(PPh_3_)_4_, (Apollo Scientific, catalog no.: OR4225, CAS: 14221-01-3, purity: >99%).

### Solvents for CP synthesis and SWCNT sorting/functionalization

The following solvents were used: toluene (Alfa Aesar, catalog no.: 19376.K2, CAS: 108-88-3, spectrophotometric grade, purity: >99.7%), DMF (Acros Organics, catalog no.: 348435000, CAS: 68-12-2, purity: 99.8%, extra dry over molecular sieve AcroSeal), methanol (Chempur, catalog no.: 116219904, CAS: 67-56-1, purity: p.a.), ethanol (Stanlab, catalog no.: 603-002-00-5, CAS: 64-17-5, purity: 96%), chloroform (Stanlab, catalog no.: 062-006-00-4, CAS: 67-66-3, purity: p.a.), acetone (ChemLand, catalog no.: 102480111, CAS: 67-64-1, purity: p.a.), iodobenzene (Alfa Aesar, catalog no.: A12788, CAS: 591-50-4, purity: 98%), bromobenzene (Acros Organics, catalog no.: A0417929, CAS: 108-86-1, purity: 99%), *p*-cymene (Thermo Fisher Scientific, catalog no.: A19226.AP, CAS: 99-87-6, purity: 97%), benzene (Chempur, catalog no.: 111625000, CAS: 71-43-2, purity: p.a.), chlorobenzene (Thermo Fisher Scientific, catalog no.: 2052185, CAS: 108-90-7, purity: ≥99%), carvacrol (Ambeed, catalog no.: A288147, CAS: 499-75-2, purity: 99.92%), *o*-xylene (Thermo Fisher Scientific, catalog no.: A11358, CAS: 95-47-6, purity: 99%), *m*-xylene (Thermo Fisher Scientific, catalog no.: L03788, CAS: 108-38-3, purity: 99%), *p*-xylene (Thermo Fisher Scientific, catalog no.: A10534, CAS: 106-42-3, purity: 99%), acetonitrile (Thermo Fisher Scientific, catalog no.: A/0620/PB08, CAS: 75-05-8, purity: ≥99%), 1,2,3,4-tetrahydronaphtalene (Thermo Fisher Scientific, catalog no.: 1467, CAS: 119-64-2, purity: ≥98%), and DMSO (Chempur, catalog no.: 113635510, CAS: 67-68-5, purity: HPLC).

### Reagents for SWCNT functionalization

The following reagents were used: PhN_2_H_3_ (Alfa Aesar, catalog no.: A11246. 18, CAS: 100-63-0, purity: 97%), aniline (Chempur, catalog no.: chem*111458308*250 ml, CAS: 62-53-3, purity: p.a.), and cholesterol (Thermo Fisher Scientific, catalog no.: 110190250, CAS: 57-88-5, purity: 95%)

### Polymer synthesis

The syntheses of PFO and PFO-BPy (table S6) were carried out in accordance with the Suzuki coupling procedure presented below. The structures of the obtained polymers were confirmed using proton nuclear magnetic resonance (^1^H NMR) spectroscopy, and their macromolecular parameters were determined with size exclusion chromatography (SEC). In brief, organoboron derivative PA [9,9-di-*n*-alkylfluorene-2,7-diboronic acid bis(pinacol)ester, purity: 98%] (0.500 g, 0.763 mmol, 1.00 mol equiv) and a sufficient amount of dibromo derivative PB (9,9-dialkyl-2,7-dibromofluorene, purity: 96%) (0.418 g, 0.763 mmol, 1.00 mol equiv) were added to the high-pressure glass reaction vessel. The reactor was then filled with 1 M Na_2_CO_3_ solution (12 ml) and toluene (12 ml). Three drops of Aliquat 336 phase transfer catalyst (PTC) were added. The mixture was purged with argon for 30 min, and subsequently, Pd(PPh_3_)_4_ (0.022 g, 0.019 mmol, 0.025 equiv) was added. The reaction mixture was stirred vigorously at 80°C for 4 days. Afterward, the reaction mixture was cooled and poured into a mixture of methanol and water in a 6:1 ratio. The primary product was then filtered off, dissolved in chloroform, and dried using MgSO_4_. The collected material was evaporated to dryness and dissolved in a sufficient volume of chloroform. The final product was precipitated in methanol. The fibrous polymer was collected by filtration, washed twice with 50 ml of cold methanol, and finally twice with 50 ml of cold acetone.

### CPE process

Three milligrams of SWCNTs and 12 mg of CP were introduced to a 19-ml glass vial. Twelve milliliters of toluene was then added to the mixture and homogenized in a bath sonicator for 15 min (Polsonic, Sonic-2, 250 W) at 5°C. Furthermore, more vigorous sonication with a tip sonotrode (Hielscher UP200St ultrasonic generator) was performed to disentangle SWCNTs and wrap them with the CP molecules for 16 min at a power of 30 W. After sonication, the obtained suspension was transferred to a 15-ml conical tube and centrifuged at 10,000 rpm (15,314*g*) for 4 min to precipitate the bundled and nonwrapped SWCNTs, as well as CP aggregates. The lump-free generated supernatant was transferred to a fresh vial and subjected to analysis by ultraviolet-visible spectroscopy and PL excitation-emission mapping.

### Functionalization of raw SWCNTs in various solvents

Raw (6,5)-enriched SWCNTs (1.5 mg; CoMoCAT, SG65i) was dispersed in 5 ml of each of the indicated solvents (table S7) using a tip-horn sonicator, and then, 2.75 mg of PhN_2_H_3_ was added to the dispersion, vortexed, and then heated for 1 hour at 80°C. After thermal functionalization, 23 mg of (2,2,6,6-tetramethylpiperidin-1-yl)oxyl (TEMPO) was added to the material to trap radicals resulting from PhN_2_H_3_ decomposition, bath sonicated, vortexed, and filtered using a Teflon membrane. Consequently, the radicals were quenched, and non-SWCNT compounds were removed. For characterization purposes, purified functionalized (6,5) SWCNTs were selectively extracted with PFO-BPy6,6′ in toluene and then analyzed by PL spectroscopy. To avoid detector saturation and to ensure the reliability of the measurements, the SWCNT concentration was set to an absorbance value of 0.5 arbitrary units (a.u.) before the measurements (measured at the *E*_11_ optical transition at ∼1000 nm) for suspensions prepared in various solvents.

Scale-up experiments depicted in fig. S2 were performed using (6,5)-enriched CoMoCAT SWCNTs. The material was weighed in three amounts (0.75, 1.5, and 6.0 mg) and then dispersed in DMSO or toluene using solvent volumes proportional to the small-scale procedure (2.5, 5.0, and 20.0 ml, respectively). The resulting suspensions were subjected to mild sonication in an ultrasonic bath for 5 min to ensure homogeneous dispersion while limiting damage to the SWCNT structure. PhN_2_H_3_ was then introduced into the systems at a final concentration of 0.55 mg ml^−1^ (1.375, 2.750, and 11.000 mg for the individual scales). The reaction mixtures were heated at 80°C for 1 hour. After completion of the reaction, TEMPO (12.00, 23.00, and 93.75 mg) was added to each sample, maintaining a final concentration of 4.6 mg ml^−1^, to quench the reactive intermediates and stabilize the system. The resulting materials were separated by filtration and then redispersed according to the CPE procedure described above.

### Functionalization of selectively extracted SWCNTs in various solvents

Raw (6,5)-enriched SWCNTs (1.5 mg; CoMoCAT, SG65i) was dispersed in the presence of 6 mg of PFO-BPy or 9 mg of PFO in 5 ml of toluene using a tip-horn sonicator to obtain near monochiral (6,5) and (7,5) SWCNT fractions. Then, toluene was evaporated, and the remaining precipitate was redispersed in a spectrum of indicated solvents using a sonication bath. Subsequently, the material was subjected to covalent modification following the previously described methodology. Once the 1-hour-long heating was complete, 23 mg of TEMPO was added to halt the progress of the reaction. The mixture was then filtered and redispersed in 5 ml of toluene solutions of PFO-BPy or PFO at 1 mg/ml concentration. The process of transferring SWCNTs back to toluene was crucial for performing the measurement, because it is not possible to maintain them in a stable state in some of the liquid media. The concentration of the SWCNTs was adjusted to 0.5 a.u. (measured at the *E*_11_ optical transition at ∼1000 nm) for suspensions prepared in various solvents.

### Determination of water content via Karl Fischer titration

To precisely quantify the residual water present in the solvents used for SWCNT functionalization, we used Karl Fischer titration using a Metrohm 915 KF Ti-Touch instrument. This method, widely regarded for its high sensitivity and selectivity toward water, was chosen to accurately assess how trace moisture influences the outcome of chemical modifications. A series of measurements was performed on untreated (nondried) solvents as well as on representative solvents that were dried using 4-Å molecular sieves for 2 weeks. The comparative analysis enabled a direct evaluation of the effectiveness of the drying procedure.

### CP removal from the SWCNT surface

In order to wash off excess CP molecules from the SWCNTs, the SWCNT dispersions were first precipitated from the solution using methanol. Then, the material was purified by membrane filtration (Ahlstrom, polytetrafluoroethylene, pore size: 2 μm, membrane thickness: 40 ± 5 μm) with hot toluene (80°C). Then, the solid was washed twice with methanol and chloroform, in which the CP dissolves very well. Last, the membrane was washed with toluene to remove the previously used solvents. The SWCNTs prepared in this way were then redispersed in toluene using a different polymer for analysis of its impact on the reactivity of SWCNTs.

### Characterization

^1^H NMR spectra of CPs (figs. S11 and S12) were registered using a Varian Unity Inova spectrometer operating at 400 MHz. ^1^H-chemical shifts were measured in δ [parts per million (ppm)], using the characteristic peak for CDCl_3_ (residual peak δ = 7.26 ppm) as a reference.

The synthesized polymers were analyzed by SEC to deduce their molecular characteristics, including molecular weights and dispersity (Ð) indices (table S8). This analysis was performed using a 1100 Agilent 1260 Infinity device from Agilent Technologies. The SEC setup comprised an isocratic pump, autosampler, degasser, a thermostatic box for columns, and a differential refractometer MDS RI Detector. Data acquisition and processing were carried out using the Addon Rev. B.01.02 data analysis software from Agilent Technologies. Calibration for SEC-calculated molecular weight was accomplished using linear polystyrene standards ranging from 580 to 300,000 g/mol. The separation of analytes was achieved using a precolumn guard (5 μm, 50 × 7.5 mm) and two columns (PLGel 5-μm MIXED-C 300 × 7.5 mm and PLGel 5-μm MIXED-D 300 × 7.5 mm). CHCl_3_ (HPLC grade) served as the solvent with a flow rate of 0.8 ml/min. The measurements were conducted at room temperature.

Optical absorption spectra of freshly collected supernatants were measured within the wavelength range of 280 to 1100 nm using a Hitachi U-2910 spectrophotometer. A double-beam mode was used with a pure solvent cuvette placed in the reference channel. The measurement was performed using 5-mm quartz cuvettes.

PL excitation-emission maps were acquired using a ClaIR microplate reader (Photonetc, Canada). The data were registered in the ranges of 460 to 900 nm (excitation) and 900 to 1600 nm (emission). The results were then visualized using OriginPro 2022b software. Whenever a solvent different than toluene was used for SWCNT processing, the material was filtered and redispersed in toluene solutions (1 mg/ml) of PFO-BPy or PFO for analysis.

Raman spectroscopy of drop-cast SWCNT samples was performed with a Renishaw inVia confocal Raman microscope in backscattering configuration and a 20× objective (Olympus MPlanFLN 20× Microscope Objective) equipped with 532- and 633-nm excitation lasers. Over 200 individual spectra were acquired and averaged for each sample.

XPS analysis was conducted in an ultrahigh vacuum experimental setup (base pressure, 8.5·10^−9^ Pa) with the use of a PREVAC EA15 hemispherical electron energy analyzer fitted with a two-dimensional (2D) MCP detector. For sample excitation, a monochromated x-ray source (dual-anode PREVAC XR-40B source, RMC50 monochromator; Al-Kα excitation line with energy 1486.60 eV) was used. Survey spectra for initial sample characterization were recorded with pass energy (PE) set to 200 eV (scanning step of 0.8 eV). For high-resolution energy regions (scanning step of 0.07 eV), PE was set to 100 eV. To minimize analyzer-induced distortions, all measurements were performed with a normal take-off angle and the curved (0.8 × 25 mm) analyzer exit slit. The binding energy scale of the analyzer was calibrated to the Au 4f_7/2_ (84.0 eV) region of the gold-covered sample, which was placed at the same sample (*102*). To avoid possible charging-related parasitic effects, the binding energy scale was additionally referenced to carbon C1s, whose energy position was determined using the procedure proposed by Greczynski and Hultman (*103*). Spectra were processed using CASA XPS software (version 2.3.25) with the application of built-in algorithms. The particular components were fitted with a combination of Gaussian (30%) and Lorentzian (70%) functions. Shirley’s function was applied for the background subtraction.

### Modeling

The SP-DFT (*81*, *82*) calculations for (6,5) SWCNT, PFO-BPy6,6′ of different lengths (1, 3, 5, or an infinite number of monomers) were carried out using a generalized gradient approximation (GGA) using the hybrid exchange and correlation functional, HSE06 (*89*, *90*), and double-ζ plus polarization numerical basis (DZP) sets, as implemented in SIESTA (*91*, *92*). These calculations relied on our implementation of hybrid functionals within the SIESTA numerical package, where the Hartree-Fock exchange energy is evaluated by expanding numerical atomic orbitals (NAOs) as linear combinations of Gaussian-type orbitals (GTOs). Gaussian fitting was performed using the Levenberg-Marquardt algorithm. In our simulations, we used six Gaussian functions for both the first and second ζ of the s orbitals (for H, C, and N), five Gaussians for the first and second ζ of the p orbitals (for C and N), and four Gaussians for the first ζ of the d orbitals (for C and N). For hydrogen, which includes only a single-ζ p orbital in the DZP basis, five Gaussians were used. Calculations incorporating solvent effects via the COSMO continuum solvation model (*83*, *84*) were performed using the QuantumATK numerical package (*93*, *94*) using a medium basis set. The DFT calculations for finite polymers were performed in the “molecule configuration” mode without any periodic boundary conditions being applied. The Brillouin zone was sampled only at the Γ point, while the density mesh cutoff for the real-space integrals was set at 350 Ry. For infinite polymers and SWCNTs, calculations were carried out in the “bulk configuration” mode with 1D periodic boundary conditions applied. Consequently, the sampling of the Brillouin zone was increased to (1 × 1 × 3) and (1 × 1 × 7) *k*-points in the Monkhorst-Pack scheme. All the structures were relaxed until the maximum force acting on any atom was lower than 0.004 eV/Å and the maximum stress changed by less than 0.001 GPa, while the self-consistent field (SCF) cycle was iterated until the total energy changed by less than 10^−5^ eV, and the density matrix elements differed by less than 10^−5^ per iteration. The solvent was not included in these calculations.

To explore the interactions between [PFO-BPy6,6]_5_ and (6,5) SWCNT, we conducted a series of MD and TFMC simulations (*85*, *86*). These simulations modeled infinite SWCNTs interacting with the polymer in toluene, acetonitrile, and DMSO solutions (fig. S7). Due to the use of 3D periodic boundary conditions, the simulation boxes included only two units of the (6,5) SWCNT. The PFO-BPy6,6′_5_ polymer was initially positioned near the SWCNT, aligned along its axis of symmetry. In additional simulations, three PFO-BPy6,6′_5_ polymer chains were placed around the SWCNT and distributed approximately evenly along its surface. The simulation boxes, with dimensions of 4.7 nm by 4.7 nm by 8.4 nm along the *X*, *Y*, and *Z* axes, respectively, were large enough to prevent direct interactions between periodic images of the SWCNT-polymer complex. Each box was filled with one of three solvents using Packmol (*104*): toluene (900 molecules for one polymer or 850 molecules for three polymers), acetonitrile (2046 or 2276 molecules), and DMSO (1196 or 1100 molecules). MD/MC calculations were performed using a full periodic table bonded valence forcefield—a Universal Force Field (UFF) potential (*105*) as implemented in QuantumATK (*94*, *106*). Energy contributions to the UFF potential were represented by simple functions based on bond lengths, bond angles, torsion angles, inversion angles, and interatomic distances. The electrostatic interactions were calculated using the smooth-particle-mesh-Ewald (SPME) solver (*107*). The cutoff used for calculating the real-space interactions was set to 7.5 Å, while the relative accuracy of SPME summation was set to 0.0001. Atomic partial charges on each atom were assigned using the QEq charge equilibration method (*108*). Dispersive interactions were included in the form of a Lennard-Jones potential (*109*, *110*) with a 10-Å cutoff and a 2-Å smoothing length. The production NPT simulations were preceded by short equilibration runs using the NPT ensemble and a Berendsen thermostat (*111*) at 300 K. Initial atomic velocities were randomly assigned based on the Maxwell-Boltzmann distribution. The thermostat relaxation time was set to 100 fs, and the simulations were performed with a time step of 0.02 fs over a duration of 5 ps (500,000 steps). These equilibration runs were followed by 20 ps (2,000,000 steps) of NPT simulations at 300 K and 1 bar, using a Martyna-Tobias-Klein barostat and thermostat (*112*). The thermostat and barostat relaxation times were set to 500 and 1000 fs, respectively, with the time step maintained at 0.02 fs. Following the production MD simulations, a brief geometry optimization was performed using the LBFGS algorithm for 10,000 steps (*113*). The system was then equilibrated under microcanonical (NVE) conditions at 300 K for 5 ps (50,000 steps) using a 0.1-fs time step. This was followed by another round of geometry optimization: 2000 steps using the first-FIRE algorithm (*114*) and a final 500-step L-BFGS minimization. To extend the sampling and capture longer-timescale phenomena, we used the TFMC method implemented in QuantumATK. TFMC simulations were conducted under NPT conditions using the Berendsen thermostat and barostat at 300 K and 1 bar, over a period of 5.31 ns (4,000,000 steps) for the systems containing one polymer chain and 10 ns for the systems containing three polymer chains. The maximum atomic displacement per Monte Carlo step was set to 0.05 Å. The system compressibility was defined as 0.0005 bar^−1^ and the barostat coupling factor was set to 1000. The radial distribution functions (RDFs) and mass density profiles (MDPs) were calculated using the data obtained during the final 4 ns for the systems containing one polymer chain and the final 1 ns for the systems containing three polymer chains.

The DFTB method (*87*) was used to further analyze the differences in the interaction between PFO-BPy6,6′ and the (6,5) SWCNT. Due to computational constraints, the systems used for DFTB modeling were reduced in size compared to those used in the MD/MC simulations shown in fig. S7. Specifically, smaller subsystems were extracted from the final MD/MC snapshots by retaining an ∼8-Å thick solvent layer surrounding both the SWCNT and the polymer. These reduced configurations are illustrated in the insets of Fig. 5. We also performed DFTB calculations for a reference system in which no polymer molecules were present [2 units of (6,5) SWCNT immersed in a toluene, acetonitrile, or DMSO solution]. We used the Slater-Koster parameter set mio-1-1 (*87*, *115*, *116*) for C, N, and H atoms in systems containing toluene and acetonitrile, and the auorg-1-1 (*87*, *115*, *117*) parameter set for C, N, O, S, and H atoms in systems containing DMSO. The use of auorg-1-1 was necessary for DMSO, as the mio-1-1 parametrization resulted in incorrect molecular geometries. The DFTB calculations included a self-consistent charge correction that considered the charge fluctuations due to interatomic electron-electron interactions. Due to the size of the system, the Brillouin zone was sampled only at the Γ point. The density mesh cutoff was set to 10 Ha for real-space integrals, and the interaction maximum range was set to 10 Å. Systems were relaxed until the maximum force acting on any atom was lower than 0.05 eV/Å and the maximum stress changed by less than 0.1 GPa. The SCF cycle was iterated until the density matrix elements changed by less than 10^−5^ per iteration. During the DFTB calculations, 3D periodic boundary conditions were applied.
