# Supplementary material for: Rapid functionalization of single-walled carbon nanotubes in polar solvents
Source: Sci Adv. 2026 Sep 11;12(37):eaeb3240. doi: 10.1126/sciadv.aeb3240 (PMC13564823; doi:10.1126/sciadv.aeb3240)
Supplement: Supplementary file 1 [file sciadv.aeb3240_sm.pdf]

Science Advances
AAAS

Supplementary Materials for

Rapid functionalization of single-walled carbon nanotubes in polar solvents

Dominik Just *et al.*

Corresponding author: Dominik Just, dominik.just@polsl.pl; Dawid Janas, dawid.janas@polsl.pl

Sci. Adv. **12**, eaeb3240 (2026)
DOI: 10.1126/sciadv.aeb3240

This PDF file includes:

Supplementary Text
Tables S1 to S8
Figs. S1 to S12

Supplementary Text

S1. Spectral data extracted from the PL plots related to modification of polymer-free SWCNTs

Table S1. Estimated positions of key PL wavelengths for SWCNT products shown in Figure 1b.

Solvent	E_{11} [nm]	E_{11}* [nm]	E_{11}*- [nm]
Ref.	996	-	-
Toluene	998	1 123, 1 158	1 284
o-xylene	998	1 123, 1 158	1 284
m-xylene	998	1 123, 1 158	1 284
p-xylene	998	1 123, 1 158	1 284
p-cymene	998	1 127, 1 158	1 271
Carvacrol	998	1 130, 1 158	-
Tetralin	998	1 130, 1 154	1 277
Chlorobenzene	998	1 133, 1 158	1 284
Anisole	998	1 127, 1 158	1 281
Acetonitrile	998	1 130, 1 158	1 284
N,N-dimethylformamide	998	1 134, 1 158	1 277
Dimethyl sulfoxide	1 001	1 120, 1 158	1 254
Water	998	1 127, 1 158	1 284

S2. Analysis of polymer-free SWCNTs modified with PhN_2H_3 in selected solvents

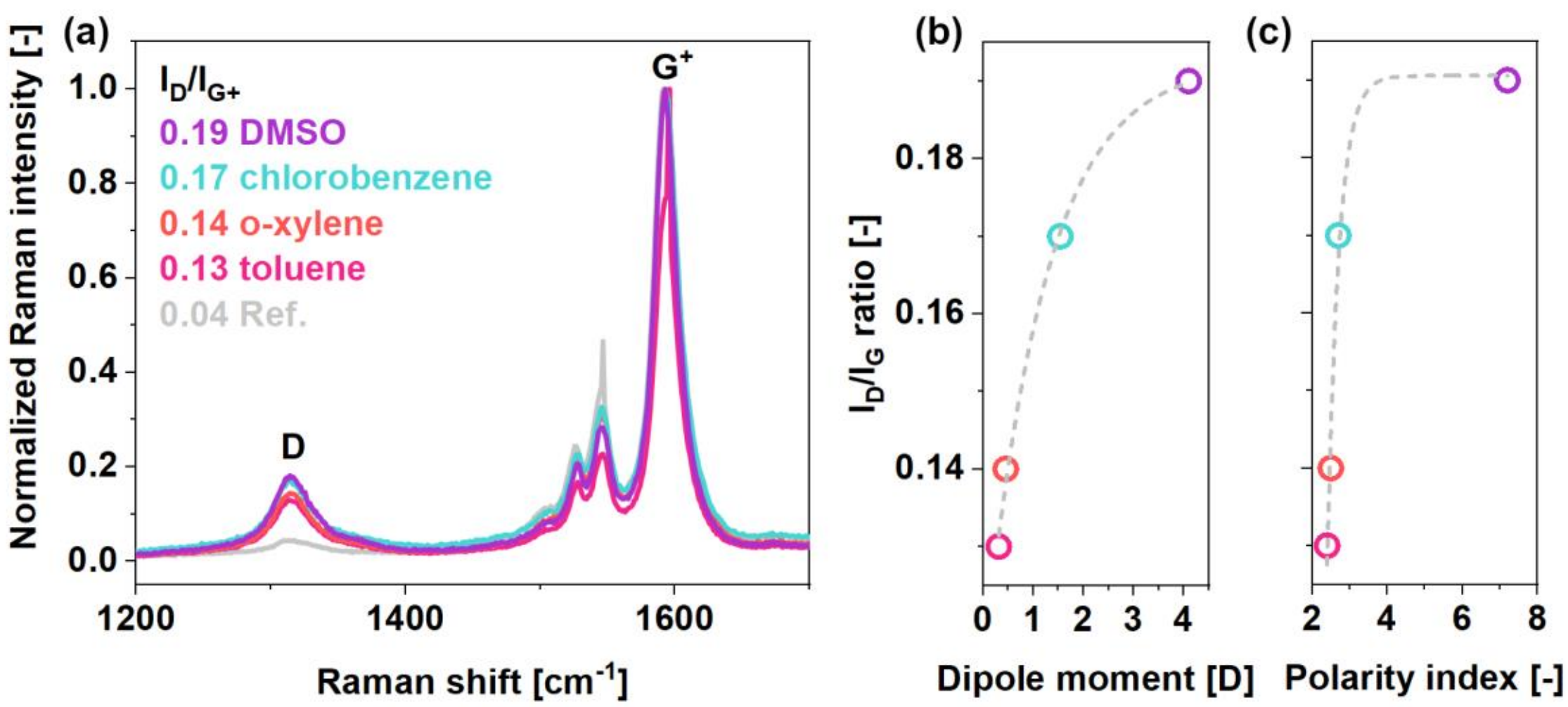

Fig. S1. Characterization of modified, polymer-free SWCNTs. (a) Raman spectra and I_D/I_G ratios of starting SWCNTs and SWCNTs covalently modified with PhN_2H_3 in toluene, *o*-xylene, chlorobenzene, and DMSO registered at the excitation wavelength of 532 nm. Exponential fits of the recorded I_D/I_G ratios with (b) dipole moments and (c) polarity indices of the indicated solvents.

S3. Scalability of SWCNT functionalization in various solvents

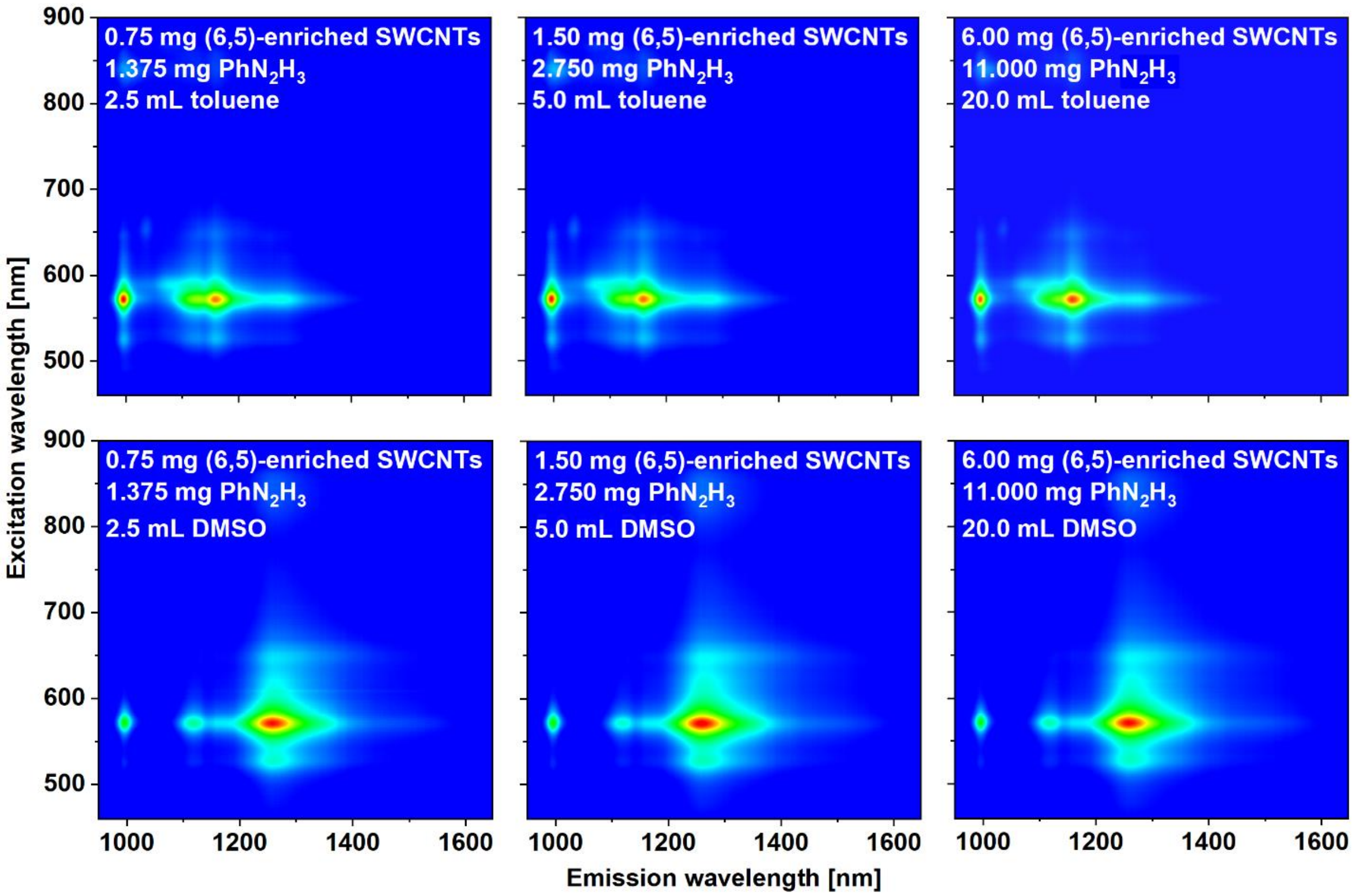

Fig. S2. PL excitation-emission maps of SWCNTs covalently modified with PhN_2H_3 in toluene and DMSO at various scales.

S4. Spectral data extracted from the PL plots related to modification of polymer-dispersed SWCNTs

Table S2. Estimated positions of key PL wavelengths for SWCNT products shown in Figure 2b.

Solvent	E_{11} [nm]	E_{11}* [nm]	E_{11}*- [nm]
Ref.	1 001	-	-
Toluene	1 005	1 158	-
o-xylene	1 005	1 158	1 294
m-xylene	1 005	1 158	-
p-xylene	1 005	1 158	-
p-cymene	998	1 158	-
Carvacrol	1 005	1 130, 1 158	-
Tetralin	1 005	1 158	1 274
Chlorobenzene	1 005	-	-
Anisole	1 005	1 158	1 287
Acetonitrile	1 005	1 130, 1 161	1 297
N,N-dimethylformamide	1 005	1 133, 1 158	1 284
Dimethyl sulfoxide	1 005	1 133, 1 158	1 267

Table S3. Estimated positions of key PL wavelengths for SWCNT products shown in Figure 2f.

Solvent	E_{11} [nm]	E_{11}* [nm]	E_{11}*- [nm]
Ref.	1 045	-	-
Toluene	1 045	1 203	-
o-xylene	1 045	1 203	-
m-xylene	1 045	1 203	-
p-xylene	1 045	1 203	-
p-cymene	1 045	1 203	-
Chlorobenzene	1 045	1 210	-
Bromobenzene	1 045	1 207	-
Iodobenzene	1 045	1 203	-

S5. Analysis of (7,5) SWCNTs modified with PhN_2H_3 in selected halogen-containing aromatic solvents

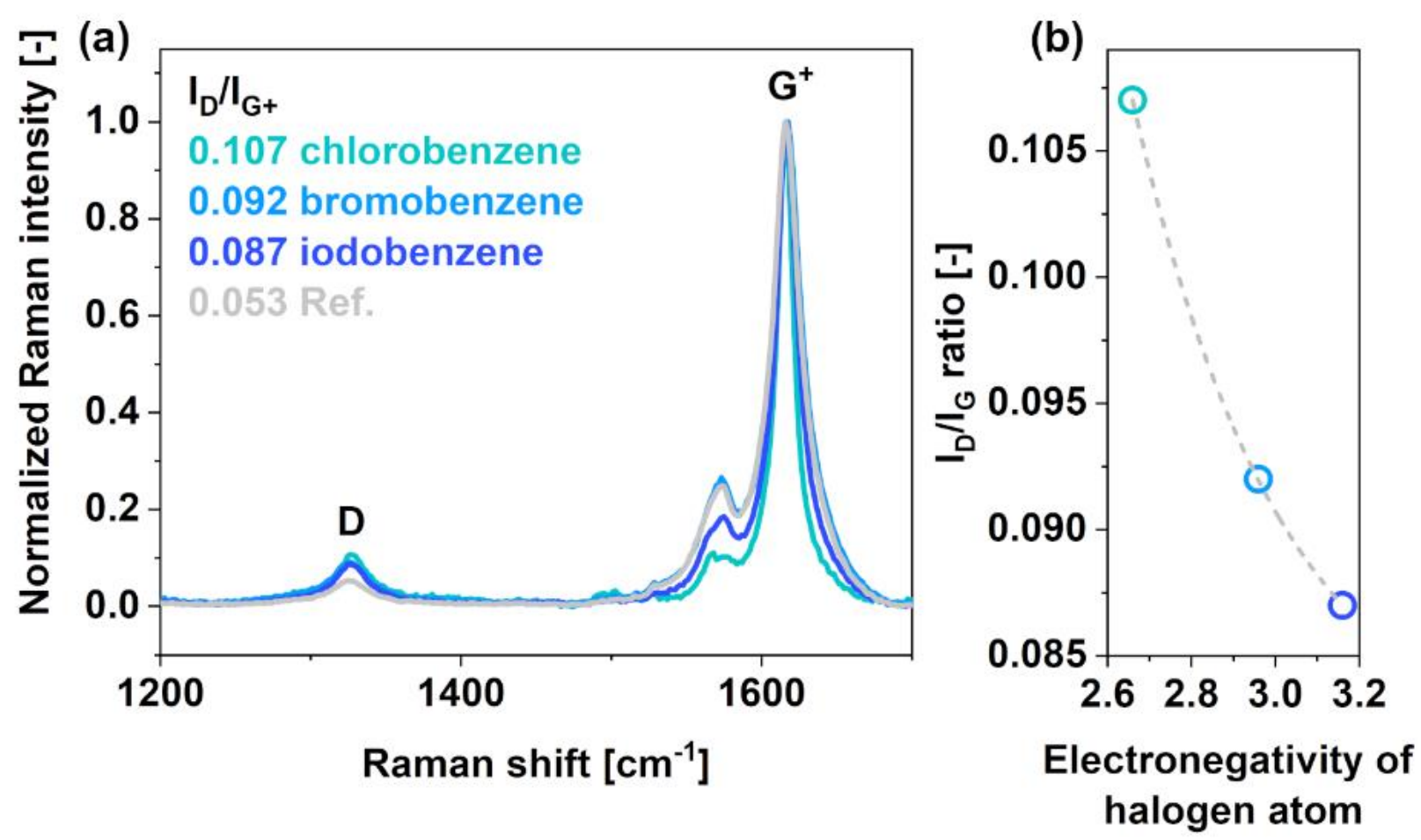

Fig. S3. Raman spectroscopy characterization of modified (7,5) SWCNTs. (a) Raman spectra and I_D/I_G ratios of starting SWCNTs and SWCNTs covalently modified with PhN_2H_3 in iodobenzene, bromobenzene, and chlorobenzene registered at the excitation wavelength of 633 nm. (b) Exponential fit of the recorded I_D/I_G ratios with electronegativity of the halogen atom.

S6. Spectral data extracted from the PL plots related to modification of post-processed polymer-dispersed SWCNTs

Table S4. Estimated positions of key PL wavelengths for SWCNT products shown in Figure 3b.

Solvent	E_{11} [nm]	E_{11}* [nm]	E_{11}*- [nm]
Acetonitrile (regular)	1 001	1 131, 1 162	1 297, 1 410
Acetonitrile (dried)	1 001	1 162	1 281, 1 356
N,N-dimethylformamide (regular)	1 005	1 137, 1 158	1 287
N,N-dimethylformamide (dried)	1 005	1 134, 1 162	1 281
Dimethyl sulfoxide (regular)	1 001	1 134, 1 158	1 267
Dimethyl sulfoxide (dried)	1 001	1 158	1 267

Table S5. Estimated positions of key PL wavelengths for SWCNT products shown in Figure 3d.

Solvent	E_{11} [nm]	E_{11}* [nm]	E_{11}*- [nm]
Toluene (CP-coated)	1 005	1 162	-
Toluene (CP-reduced)	1 001	1 162	1 353
Acetonitrile (CP-coated)	1 001	1 161	1 297
Acetonitrile (CP-reduced)	1 001	1 165	1 294
N,N-dimethylformamide (CP-coated)	1 005	1 158	1 284
N,N-dimethylformamide (CP-reduced)	1 005	1 158	1 284
Dimethyl sulfoxide (CP-coated)	1 005	1 158	1 267
Dimethyl sulfoxide (CP-reduced)	1 005	-	1 271

S7. Impact of basis sets on the spatial distribution of molecular orbitals in PFO-BPy

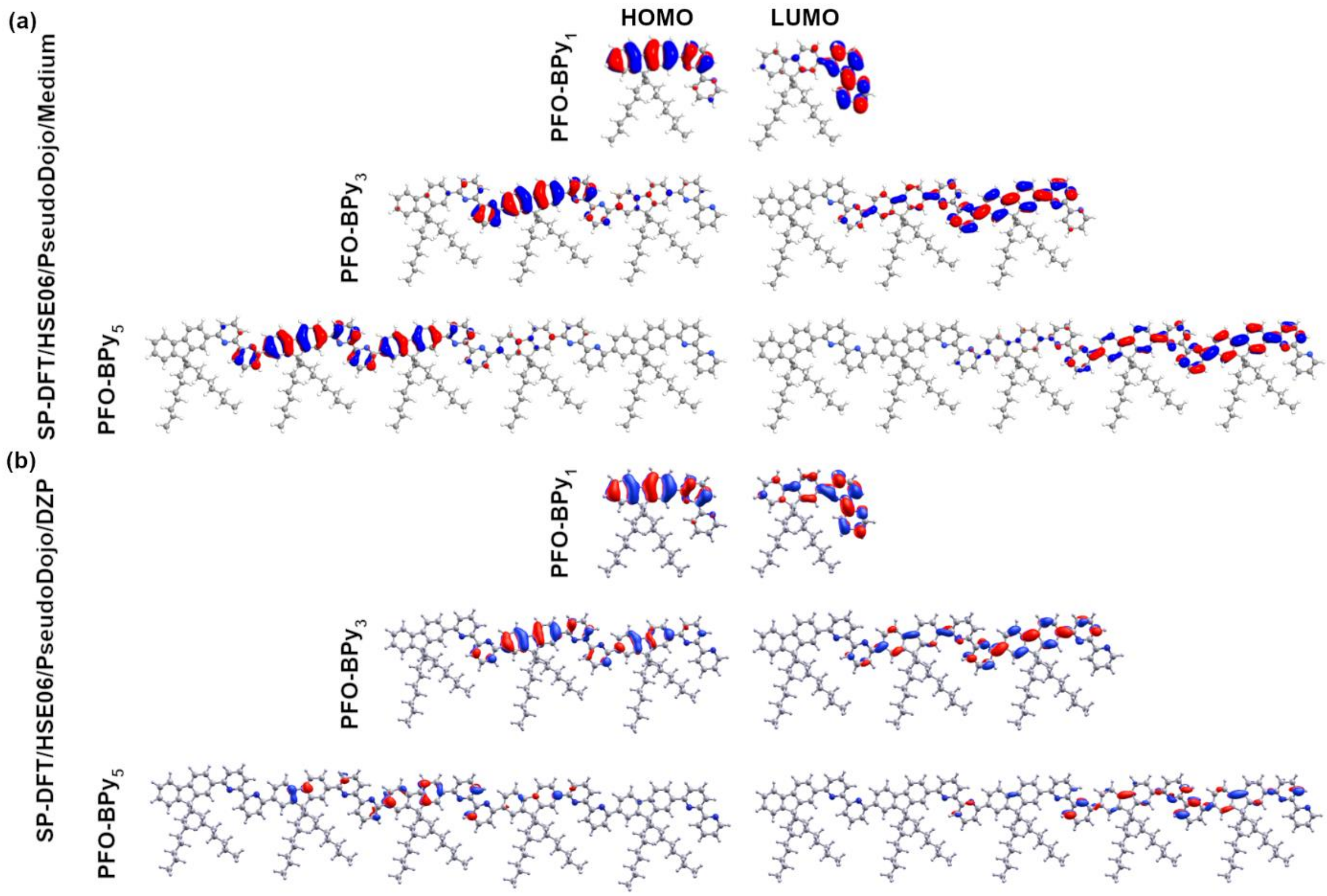

Fig. S4. HOMO and LUMO of fully relaxed PFO-BPy6,6 polymers of different lengths (1, 3, or 5 number of monomers). The data were obtained at the (a) SP-DFT/HSE06/Medium level of theory using Quantum ATK visualized at a 0.06 Å$^{-3/2}$ isovalue and (b) SP-DFT/HSE06/DZP level of theory using SIESTA visualized at a 0.036 isovalue. Positive values of wave functions are represented by blue, whereas negative values are represented by red. Carbon, nitrogen, and hydrogen atoms are marked in gray, blue, and white, respectively. Our DFT calculations showed that the PFO-BPy HOMO is mainly formed by the PFO unit and, to a lesser extent, by the BPy unit, whereas the PFO-BPy LUMO is localized only on the BPy unit.

S8. Impact of solvent on the morphology and electronic properties of PFO-BPy

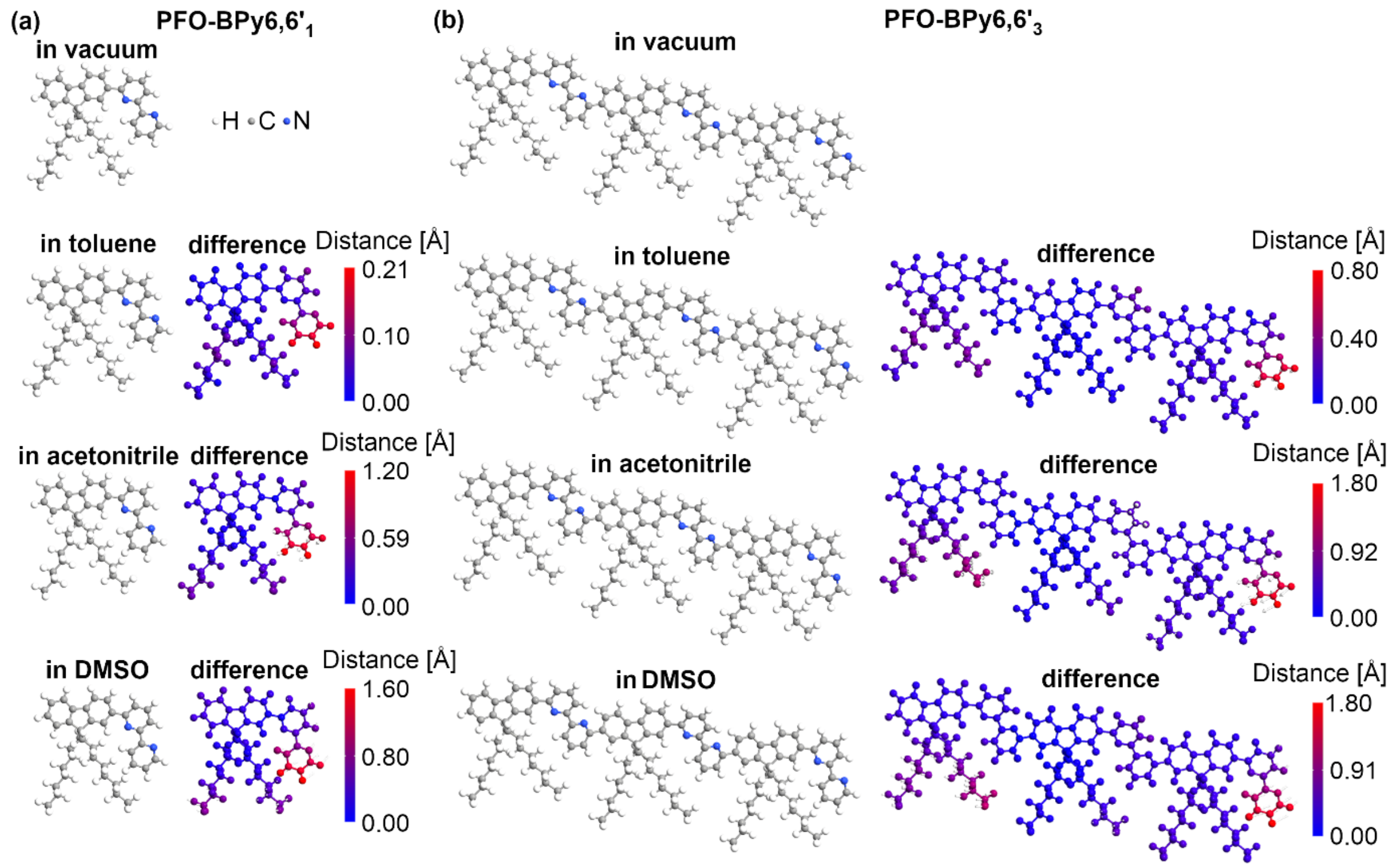

Fig. S5. Structural deviations between vacuum-optimized and solvent-relaxed geometries. (a) PFO-BPy6,6' monomer and (b) trimer calculated using the SP-DFT/HSE06/medium level of theory with the COSMO solvation model. Atom-wise displacements (in Å) relative to the vacuum configuration are shown for three solvents: toluene, acetonitrile, and DMSO. The most pronounced geometry changes are observed in the polar solvents.

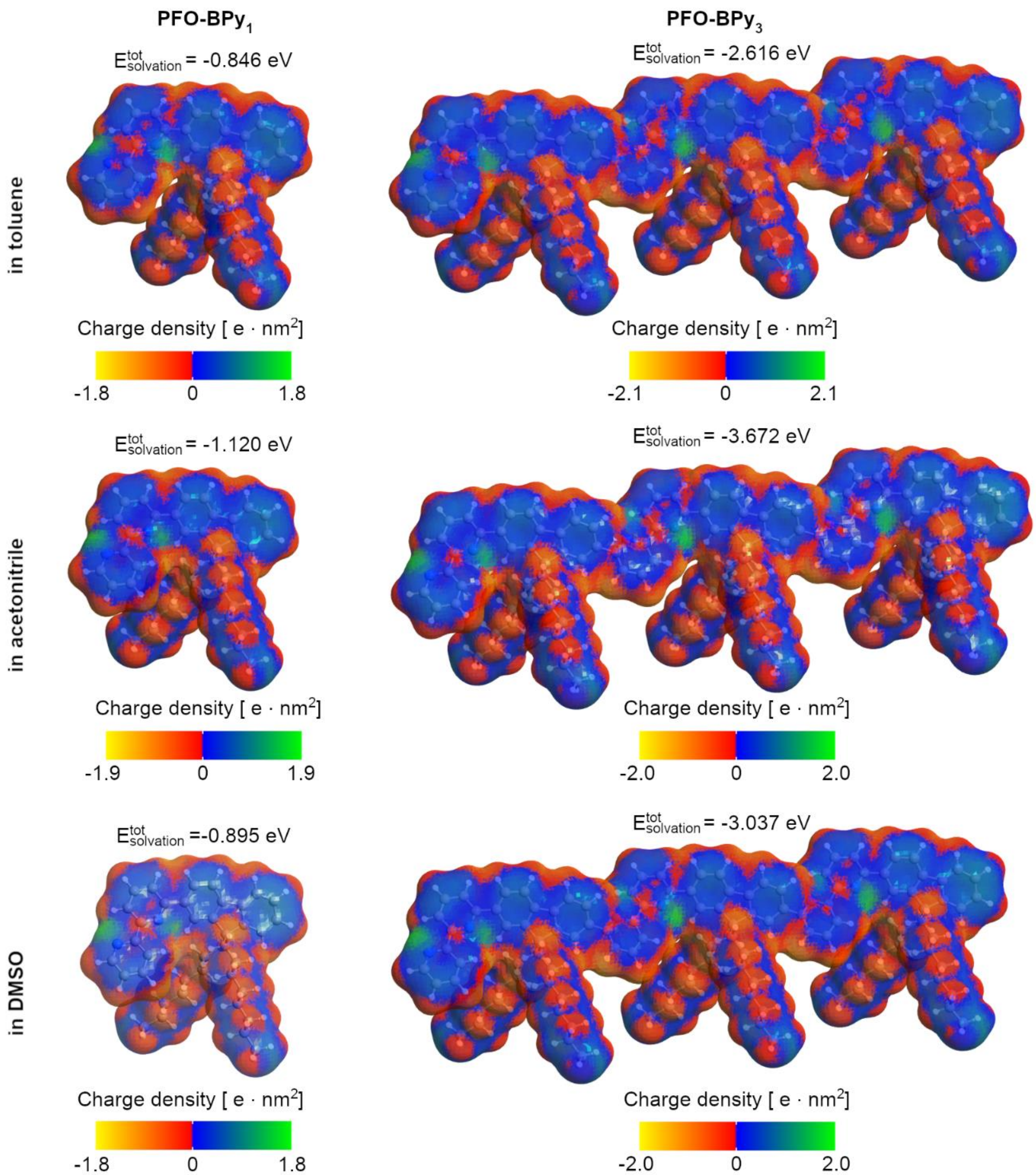

Fig. S6. Solvent-accessible surface charge distributions and corresponding solvation energies. The data relate to PFO-BPy6,6' monomer (left) and trimer (right) calculated in toluene, acetonitrile, and DMSO using the SP-DFT/HSE06/medium level of theory with the COSMO solvation model.

S9. Impact of solvent on the interaction between (6,5) SWCNT and PFO-BPy

The MD/MC modeling results presented in Figure S7 reveal subtle but consistent differences in the interactions between PFO-BPy6,6'$_5$ polymers and (6,5) SWCNTs in different solvent environments. Although the overall differences are relatively small, the radial distribution function (RDF) analysis indicates a slightly higher probability of the polymer being found in close proximity to the SWCNT surface when the system is immersed in toluene, compared to acetonitrile or DMSO (cf. the height of the first RDF peaks in Figure S7b, left panel). When three polymer chains are included, the differences between the systems become somewhat more pronounced, although they remain relatively small overall. Comparison of the SWCNT–polymer RDFs for the systems containing one polymer chain and three polymer chains shows that in toluene and acetonitrile the presence of additional polymer chains appears to shift the polymers slightly farther away from the SWCNT surface. This is reflected by the change in the relative intensities of the first and second RDF peaks. In Figure S7b (left panel), the first and second peaks are of comparable intensity for the toluene- and acetonitrile-based systems, whereas in Figure S7c (left panel) the first peak becomes visibly lower than the second one. This indicates a reduced probability of finding polymer fragments at the shortest SWCNT–polymer distances (ca. 5 Å) and suggests a slight shift of the polymer population toward larger distances from the SWCNT surface. In contrast, no analogous changes are observed for the DMSO-based system, for which the SWCNT–polymer RDF remains largely unchanged upon increasing the number of polymer chains.

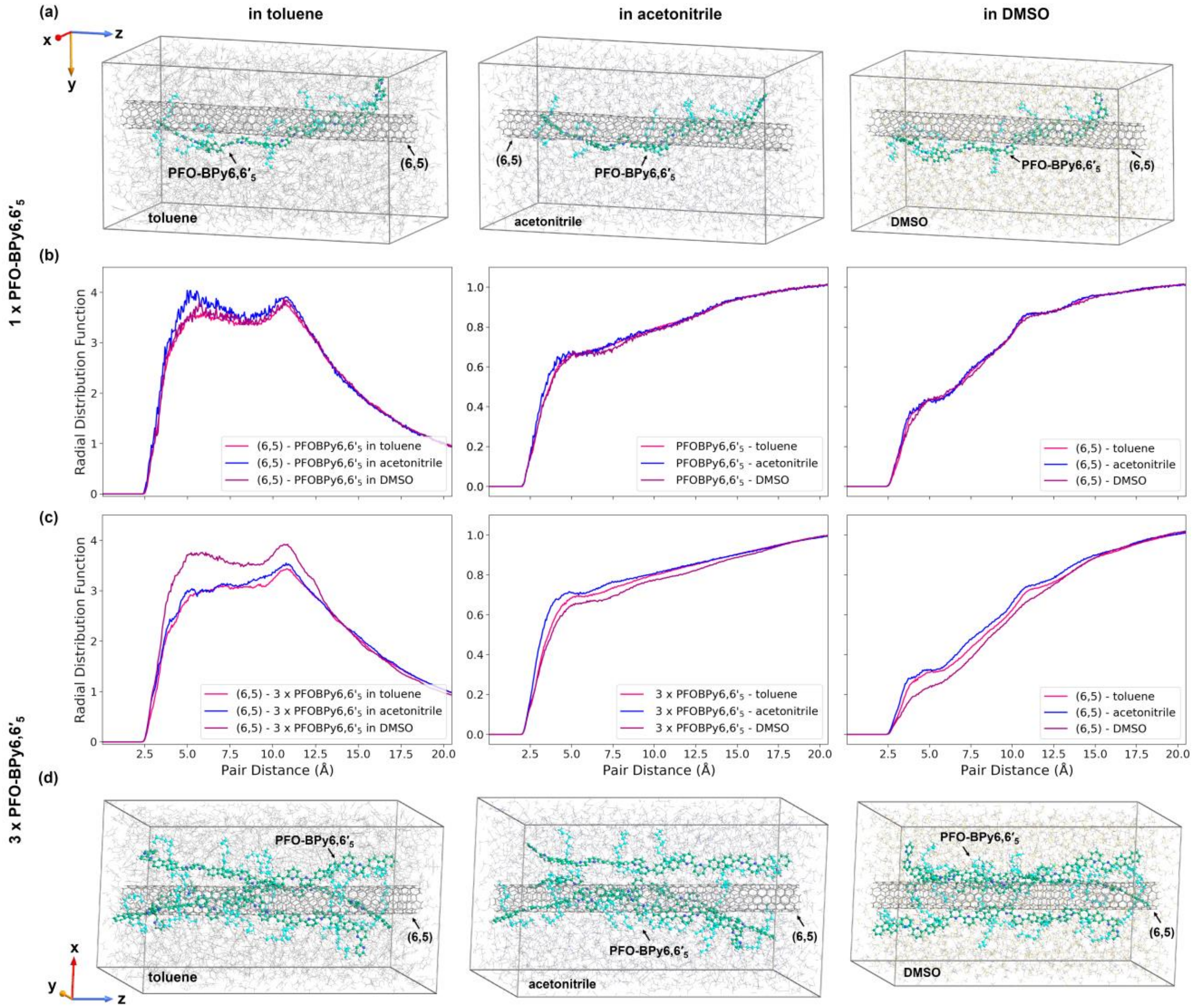

Fig. S7. MD/MC simulations of (6,5) SWCNT – PFO-BPy6,6’5 interactions in solutions. (a,d) Snapshots of the final configurations for simulation boxes containing two units of (6,5) SWCNT interacting with one (a) or three (d) PFO-BPy6,6′$_5$ polymers, each consisting of five monomers, in toluene, acetonitrile, and DMSO. SWCNTs, solvent, and polymer molecules are represented using stick, line, and ball-and-stick models, respectively. For clarity, solvent molecules are drawn with a higher degree of transparency than other system components. Carbon atoms constituting the polymer backbone and side chain are marked in teal and cyan, respectively. Other carbon atoms are grey, whereas nitrogen, oxygen, sulfur, and hydrogen atoms are blue, red, yellow, and white, respectively. (b,c) RDFs for the corresponding systems containing one polymer chain (b) and three polymer chains (c): SWCNT–polymer (left), polymer–solvent (middle), and SWCNT–solvent (right).

A more detailed RDF decomposition (Figure S8) shows that, in the systems containing one polymer chain, the polymer backbone resides closer to the SWCNT surface than the side chains in all solvents, suggesting preferential backbone–SWCNT interactions. When three polymer chains are present, this trend changes for the systems in toluene and acetonitrile. In these cases, the polymer backbone shifts slightly farther away from the SWCNT surface, whereas the side chains remain at approximately the same distance as in the corresponding one-polymer systems. As a result, the side chains are closer to the SWCNT surface than the polymer backbone. In contrast, no analogous change is observed for the DMSO-based system. Additional RDF analysis further indicates that acetonitrile molecules tend to be slightly closer to both the polymer (Figure S7b,c, middle panels) and the SWCNT surface (Figure S7b,c, right panels) than molecules of toluene or DMSO. A more accurate description of such interactions may require reactive force fields like ReaxFF or machine learning-based interatomic potentials, or higher-level methods such as DFTB or DFT, the results of which we present in the main text.

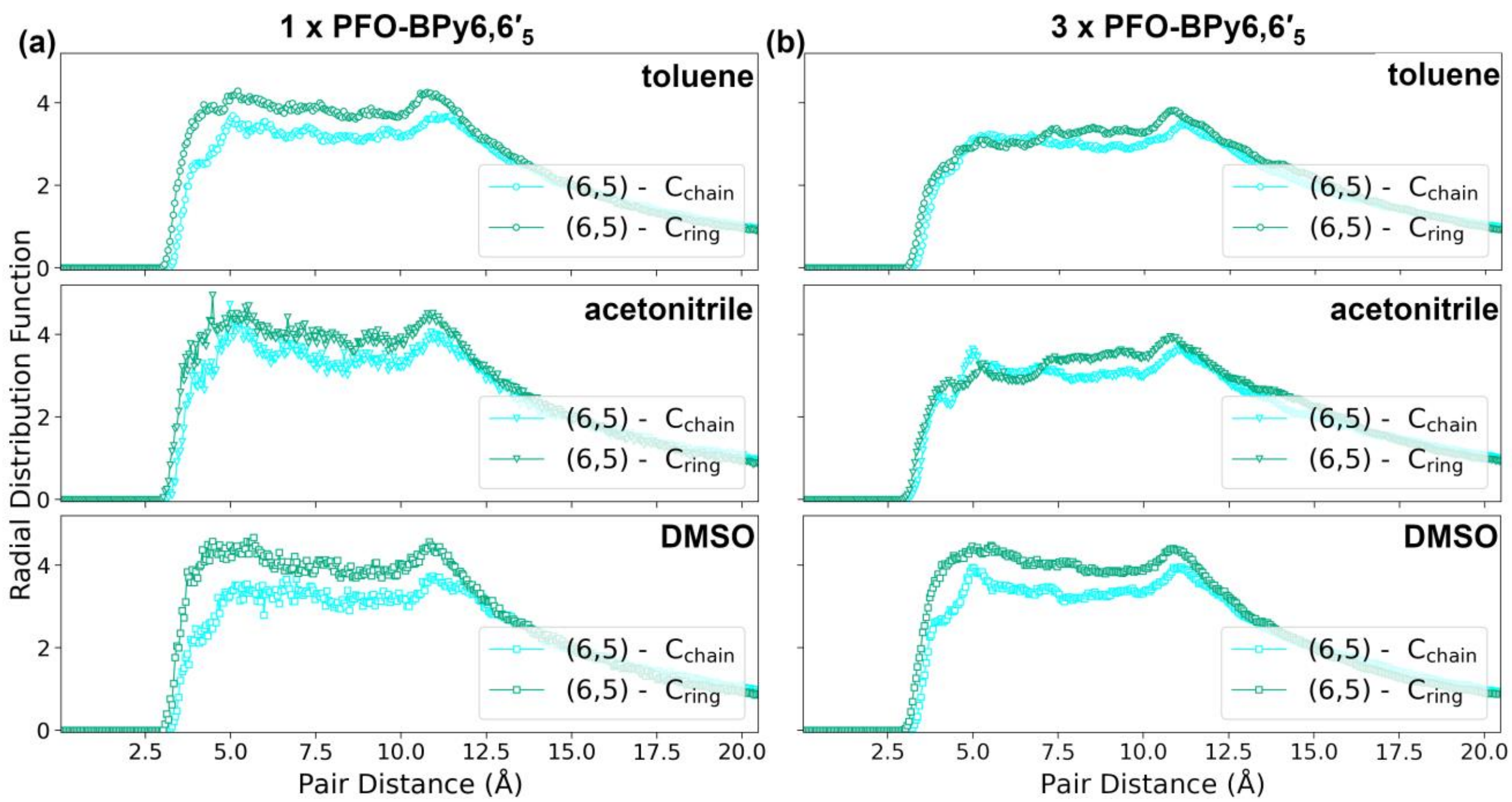

Fig. S8. RDFs of (6,5) SWCNT carbon-polymer backbone carbon (C_{ring}), SWCNT carbon-polymer side chain (C_{chain}) atom pairs. Systems containing (a) one or (b) three polymers in three different solvents.

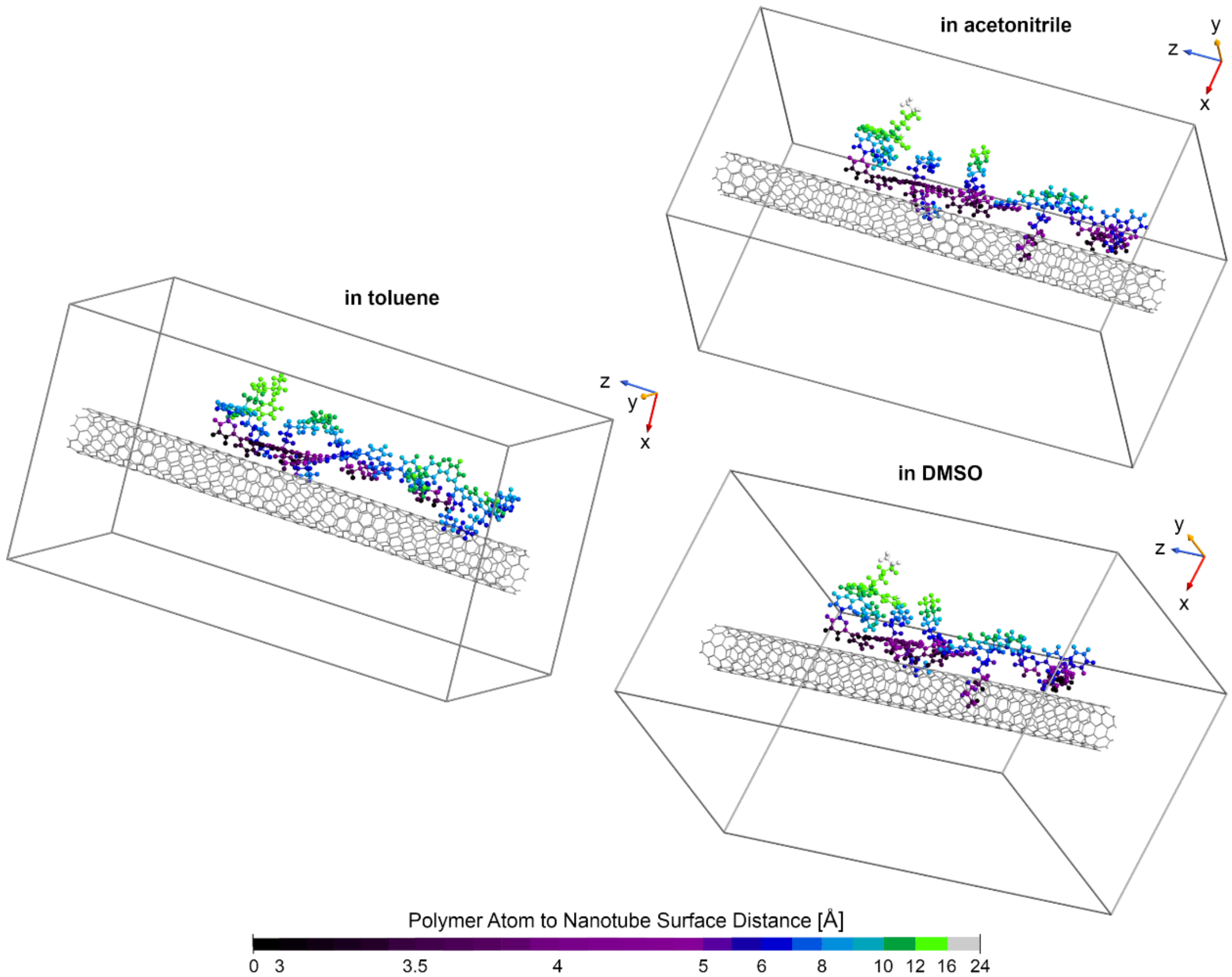

Fig. S9. Atomistic views of (6,5) SWCNT interacting with PFO-BPy6,6'$_5$ polymer in toluene, acetonitrile, or DMSO, obtained at the DFTB level. SWCNTs and polymers are shown using stick and ball-and-stick models, respectively. Solvent molecules are omitted for clarity. Polymer atoms are colored based on their distance from the SWCNT lateral surface, with darker shades indicating closer proximity and lighter shades indicating greater distance.

S10. Impact of cholesterol on the optical properties of (6,5) SWCNTs modified in toluene

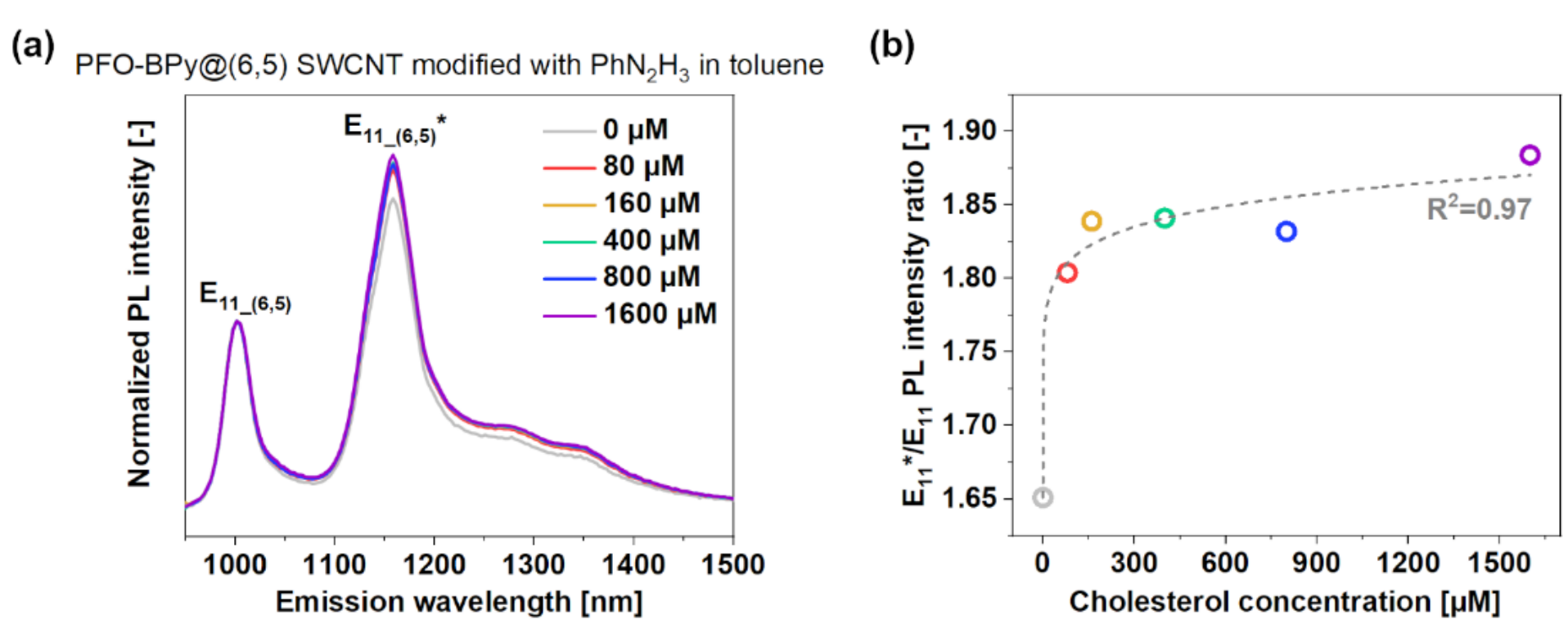

Fig. S10. Optical sensing of cholesterol using modified SWCNTs. (a) E_{11}-normalized PL spectra (λ_{ex}=579 nm, solvent: toluene) of (6,5) SWCNTs wrapped with PFO-BPy, functionalized with PhN_2H_3 in toluene, and exposed to the indicated concentrations of cholesterol, (b) relationship between E_{11}^*/E_{11} ratio and cholesterol concentration.

S11. Experimental details

Table S6. List of obtained CPs with the various subunits used for the synthesis.

Name	Acronym	PA	PB	CP no.
Poly((9,9-dioctylfluorenyl-2,7-diyl)-alt-(6,6'-(2,2'-bipyridine)))	PFO-BPy	9,9-di-n-octylfluorene-2,7-diboronic acid bis(pinacol)ester	6,6'-dibromo-2,2'bipyridine	P1
Poly(9,9'-dioctylfluorenyl-2,7-diyl)	PFO		9,9-dioctyl-2,7-dibromofluorene	P2

Table S7. List of solvents with their physical properties used in SWCNT modification

Solvent	Dipole Moment [D]	Density [g/mL]	Dielectric Constant [F/m]	Boiling Point [°C]	Viscosity at 20°C [mPa·s]
Benzene	0.00	0.876	2.27	80.1	0.61
Toluene	0.36	0.867	2.38	110.6	0.59
o-xylene	0.62	0.880	2.56	144.4	0.80
m-xylene	0.37	0.864	2.31	139.1	0.64
p-xylene	0.00	0.861	2.27	138.3	0.60
p-cymene	0.00	0.857	2.72	177.0	0.85
Carvacrol	1.70	0.977	9.80	237.7	6.10
Tetralin	0.61	0.970	2.32	207.0	2.35
Chlorobenzene	1.54	1.106	5.62	132.0	0.80
Anisole	1.38	0.995	4.33	154.0	1.30
Acetonitrile	3.92	0.786	35.9	81.6	0.36
Dimethylformamide	3.82	0.944	36.7	153.0	0.80
Dimethyl sulfoxide	3.96	1.100	47.2	189.0	2.00
Water	1.85	0.998	78.5	100.0	1.00

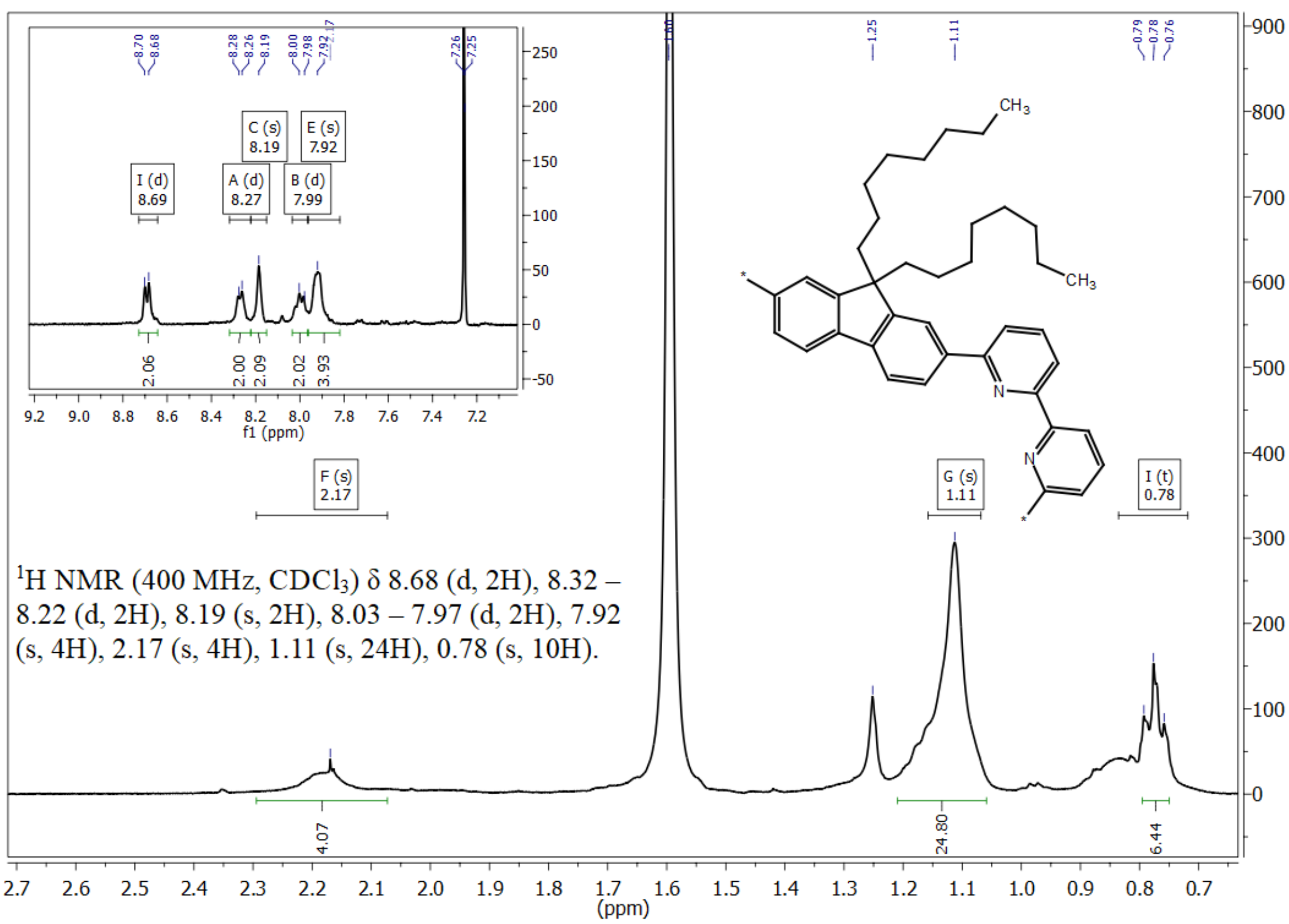

Fig. S11. ^{1}**H NMR spectrum of PFO-BPy synthesized in-house.**

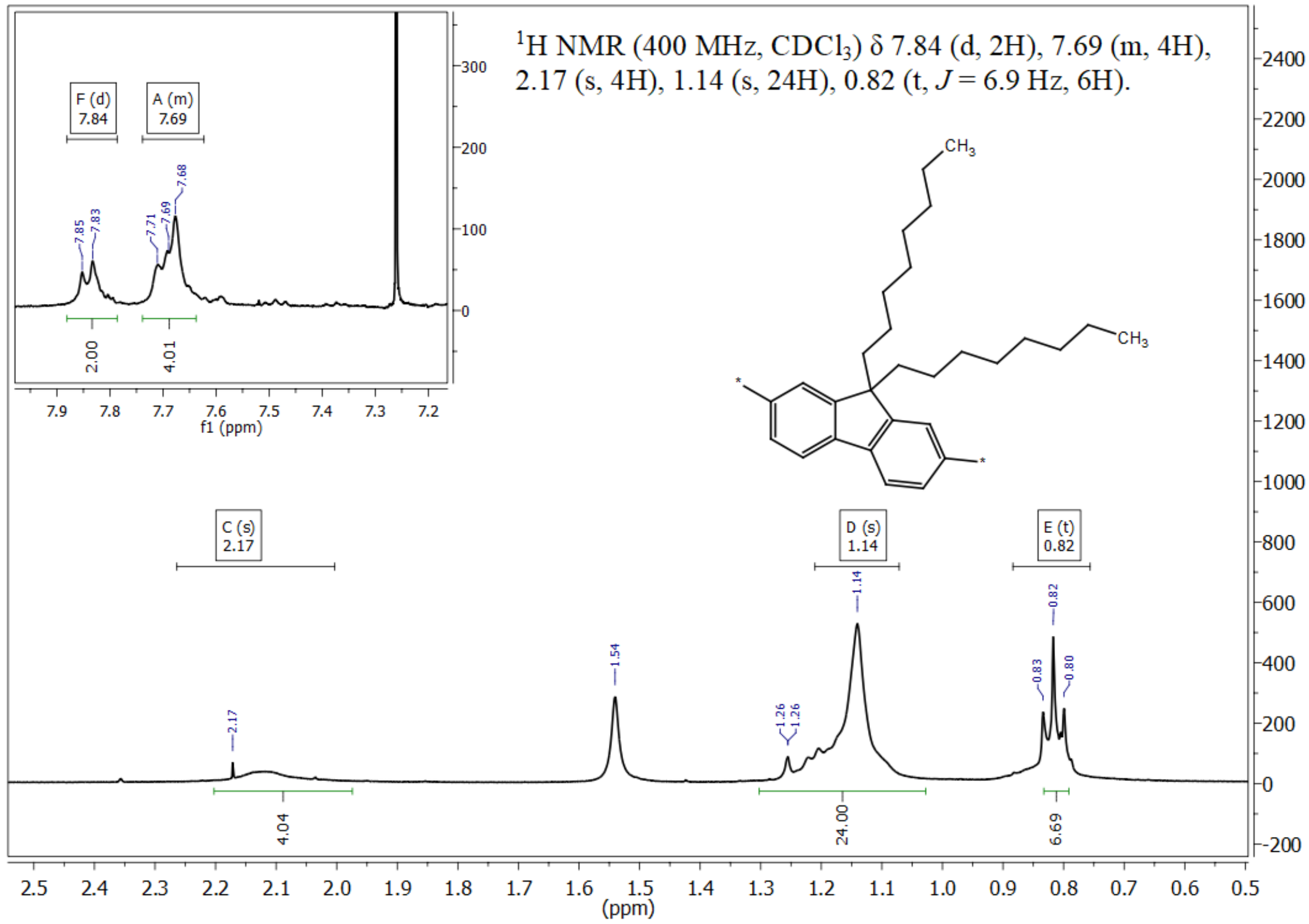

Fig. S12. ^{1}H NMR spectrum of PFO synthesized in-house.

Table S8. List of obtained CPs with the various subunits used for the synthesis.

Acronym	Polymer no.	M_n [g/mol]	M_w [g/mol]	Đ
PFO-BPy	P1	8 300	11 650	1.40
PFO	P2	23 600	53 400	2.26
